# Metamaterial based on an inverse double V loaded complementary square split ring resonator for radar and Wi-Fi applications

**DOI:** 10.1038/s41598-021-01275-6

**Published:** 2021-11-05

**Authors:** Md. Rashedul Islam, Mohammad Tariqul Islam, Mohamed S. Soliman, Mohd Hafiz Baharuddin, Kamarulzaman Mat, Asraf Mohamed Moubark, Sami H. A. Almalki

**Affiliations:** 1 Department of Electrical, Electronic and Systems Engineering, Faculty of Engineering and Built Environment, Universiti Kebangsaan Malaysia, Bangi, 43600 Selangor, Malaysia; 2 Department of Electrical Engineering, College of Engineering, Taif University, P.O. Box 11099, Taif, 21944 Kingdom of Saudi Arabia; 3 Department of Electrical Engineering, Faculty of Energy Engineering, Aswan University, Aswan, 81528 Egypt

**Keywords:** Condensed-matter physics, Engineering, Physics

## Abstract

In this research paper, an inverse double V loaded complementary square split ring resonator based double negative (DNG) metamaterial has been developed and examined numerically and experimentally. The electromagnetic (EM) properties of the proposed inverse double V-structure were calculated using computer simulation technology (CST-2019) and the finite integration technique (FIT). The designed metamaterial provides three resonance frequencies are 2.86, 5, and 8.30 GHz, covering S-, C-, and X-bands. The total size of the recommended unit cell is 8 $\times$ 8 $\times$ 1.524 mm^3^, and a high effective medium ratio (EMR) value of 13.11 was found from it. The − 10 dB bandwidths of this structure are 2.80 to 2.91, 4.76 to 5.17, and 8.05 to 8.42 GHz. The proposed structure's novelty is its small size, simple resonator structure, which provides double negative characteristics, high EMR, maximum coverage band, and required resonance frequencies. Wi-Fi network speeds are generally faster when frequencies in the 5 GHz band are used. Since the proposed structure provides a 5 GHz frequency band, hence the suggested metamaterial can be used in Wi-Fi for high bandwidth and high-speed applications. The marine radars operate in X-band, and weather radar works in S-band. Since the designed cell provides two more resonance frequencies, i.e., 2.86 GHz (S-band) and 8.30 GHz (X-band), the proposed metamaterial could be used in weather radar and marine radar. The design process and various parametric studies have been analyzed in this article. The equivalent circuit is authenticated using the advanced design system (ADS) software compared with CST simulated result. The surface current, E-field, and H-field distributions have also been analyzed. Different types of array structure, i.e., 1 $\times$ 2, 2 $\times$ 2, 3 $\times$ 3, 4 $\times$ 4, and 20 $\times$ 25 is examined and validated by the measured result. The simulated and measured outcome is an excellent agreement for the inverse double V loaded CSSRR unit cell and array. We showed the overall performance of the suggested structure is better than the other structures mentioned in the paper. Since the recommended metamaterial unit cell size is small, provides desired resonance frequency, gives a large frequency band and high EMR value; hence the suggested metamaterial can be highly applicable for Radar and Wi-Fi.

## Introduction

Metamaterial is a man-made composite material with unique features not found in nature^1^. This structure consists of exotic effects and has inspired countless researchers to build an innovative contribution direction in almost all fields^2^. A single negative (SNG) structure has negative permeability or permittivity, but a double negative (DNG) or left-handed (LH) structure has both negative permittivity and permeability. In 1968, the principle of left-hand metamaterials was first explored by the Russian physicist Victor Veselago in1967^3^. This unusual material can monitor the propagation of light when passed in waveguides and free space. As a result, electromagnetic (EM) wave manipulation has a wide range of applications, including holography, signal multiplexing, data processing, and so on. The MTM can be used in a variety of applications, including antenna design^4,5^, filters^6^, specific absorption rate (SAR) reduction^7^, invisibility cloaking^8^, superlenses^9^, absorber^10,11^, sensors^12,13^ etc. In addition, the MTM is also widely used in radar and satellite communication^14,15^.

Islam et al., proposed a new metamaterial architecture in^16^ for the satellite antenna. The defined MTM scheme was put on FR-4 substrate. The frequency spectrum 2 to 4 GHz was used for this simulation procedure. Shahidul et al., presented a crossed-line CSRR based epsilon negative (ENG) metamaterial in^17^ for tri-band microwave functions. The size of this MTM unit cell is 10 $\times$ 10 mm^2^, and the EMR is low, only 4.5. Hossain et al. recommended a meta-atom in^18^ for an effective medium ratio (EMR) that would follow the meta-atom criteria and is applicable for multiband. The size is 12 × 12 × 1.6 mm^3^, and EMR is 10.55 of this MTM. Hasan et al., developed a DNG metamaterial in^19^, which is compactly miniaturized, modified Z-shaped and applicable for wideband operation. This MTM has only one frequency band (X- band), and EMR is low, only 4. Islam et al., presented an SNG metamaterial in^15^ for the application of gain enhancement of satellite and radar antenna. It has another feature i.e., near-zero indexes, the unit cell size and ERM are 8 × 8 mm^2^ and 14.37, respectively. in^20^, Cheng et al., introduced a seven-band metamaterial absorber that is ultra-thin, compact, and has a single resonator structure and has numerous prospective applications such as detection, sensing, and imaging. Chen et al., proposed single-layer graphene tunable broadband THz MTM absorber in^21^, which has important applications in tunable filtering, sensing, and modulators. Hossain et al., proposed a composite DNG metamaterial in^22^ for multiband applications. The presented MTM unit cell shape is double C, and its EMR is moderate. Zhou et al. developed a DNG metamaterial in^23^, which is a double Z-shape, its EMR is 4.80, and size is 8.5 × 8.5 mm^2^. Hoque et al., demonstrated a DNG metasurface absorber in^24^ for dual-band applications. The size of the metasurface unit cell is large, but EMR is low. Hossain et al., described an SRR based ENG metamaterials^25^ for multiband microwave functions. Islam et al. suggested an “H-shape” DNG metamaterial unit-cell in^26^ which size was very large 30 × 30 mm^2^, but EMR was very low, only 3.65. Cheng et al., presented a broadband MTM microwave absorber in^27^, which is an asymmetric and sectional resonator and it is applicable for energy harvesting and stealth technology. Kalraiya et al., designed a polarization-independent MTM absorber in^28^ for wideband applications. The unit cell size is 12.5 $\times$ 12.5 mm^2^, which covers C-, and X- band. Zhou et al. described a 12 × 12 mm^2^ size of an SNG metamaterial unit cell in^29^ which was ‘‘S-shape’’, applicable for X- and Ku- bands and EMR was below 4. Islam et. al., also described an MTM sensor in^30^ for showing the sensitivity of the structure. The structure of metamaterial is meanderline, its sensitivity and EMR are -3 dB/mm and 7.2, respectively.

Thummaluru et al., developed an MTM absorber in^31^, which shape is four-fold symmetric, applicable only for C-band, and the cell size is very large 28.2 $\times$ 28.2 mm^2^. Faruque et al., developed a DNG metamaterial in^32^ for dual-band microwave applications, but the unit cell structure is very large 25 $\times$ 20 mm^2^. Islam et al., introduced a hexagonal SRR based MTM in^33^ for S-, and X-band applications. Its size is 10 $\times$ 10 mm^2^, and the EMR of this structure is 8.40. Rao et al., presented an MTM inspired circularly polarized antenna^34^ for WiMAX and WLAN applications. The coverage band, EMR and size of the structure are 4, 9.95, and 9 × 9 mm^2^. The MTM was used in the coplanar waveguide (CPW) antenna to increase the bandwidth, but the antenna's gain is extremely low. Cheng et al., also presented a dual-band anisotropic metamaterial in^35^, which is circular polarization and applicable for radar, satellite, and remote sensing. Zhou et al., demonstrated a high gain and wideband patch antenna in^36^ using reflective focusing metasurface. The patch antenna's initial design consists of a slot planar patch radiating portion fed by a coplanar waveguide (CPW). The simulated average gain relative bandwidth is 4.5 dBi and 76%, respectively. Hossain et al., described an MTM^37^ for a multiband meta-atoms which is left-handed obeying EMR, but its size is 11 × 10 mm^2^. Hossain et al., developed a DNG metamaterial in^38^ for dual-band microwave applications. The shape of this design is “Modified H”, and its size is 9 $\times$ 9 mm^2^. Kumari et al. demonstrated a polarization-insensitive MTM absorber in^39^, compact ultra-thin and applicable simply for X-band. The size of the MTM absorber is 10 $\times$ 10 mm^2^, and EMR is very low. Almutairi et al. demonstrated a DNG metamaterial in^40^ whose size is 5.5 × 5.5 and applicable only for C-band and EMR is 7.44. Thummaluru et al. also presented a tunable MTM absorber in^41^, a wide-angle polarization controllable and circular sector. This structure is applicable only for C-band and it’ size is 9 $\times$ 9 mm^2^. Rao et al., presented a circular-shaped metamaterial in^42^, which is applicable for multiband. This MTM was utilized to increase bandwidth in a CPW fed antenna, although the gain is relatively modest.

A DNG metamaterial has been developed by using an inverse double V inspired CSSRR resonator shape in this paper. This DNG MTM provides three resonance frequencies of 2.86, 5, and 8.30 GHz. The first resonance frequency, 2.86 GHz, covers S-band (2 to 4 GHz), the second resonance, 5 GHz, covers the C- band (4 to 8 GHz), and the third resonance, 8.3 GHz, covers X-band (8 to 12 GHz). The S-band is utilized in traffic control radar at airports, weather radar, and surface ship radar. The majority of marine, civil, military radars work on X- bands. The C-band, especially the 5 GHz band, is widely used in Wi-Fi for high bandwidth and high-speed applications. The DNG region has been found from 2.82 to 2.89, 5.04 to 5.23, and 8.36 to 8.46 GHz. The effective medium parameters, equivalent circuit, surface current, E-field, and H- field have been analyzed. Different parametric studies and arrays such as 1 $\times$ 2, 2 $\times$ 2, 3 $\times$ 3, 4 $\times$ 4, and 20 $\times$ 25 have also been examined. The proposed inverse double V-shaped DNG metamaterial has an EMR value of 4.5, which defines smallness and appropriateness.

## Design structure of the MTM unit cell

**Figure 1:**
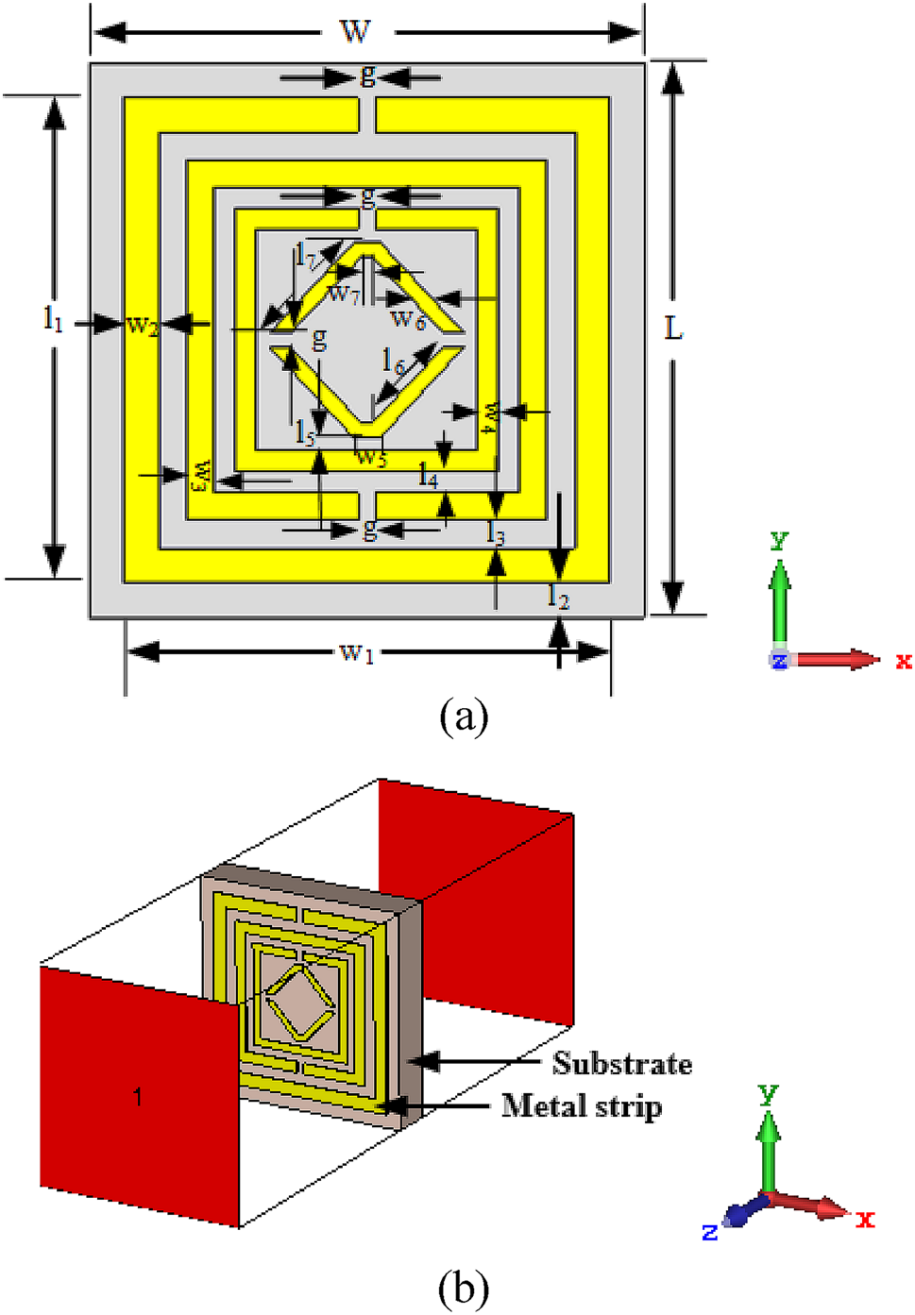
(**a**) CST simulated top view, (**b**) Perspective view of the suggested structure. (CST STUDIO SUITE 2019, https://www.3ds.com/products-services/simulia/products/cst- studio-suite**)**^43^.

Figure 1a reveals the top view of the introduced inverse double V structure, imported from CST software^43^. Firstly, the presented structure consists of Rogers RO4350B dielectric substrate material of 8 $\times$ 8 mm^2^ (length and width) with a thickness of 1.524 mm. The dielectric constant $(\varepsilon$) and tangent loss (δ) of this material are 3.66 and 0.0037. The proposed structure comprises three square rings, which are complementary split-ring resonators and inside the inverse double V-shape resonator. These resonators consist of copper which thickness was 0.035 mm. The conductivity (σ) of the resonator is 5.8 × 10^7^ S/m, which is annealed copper. The width of the first square ring is 0.4 mm, the second square ring is 0.30 mm, and the third one is 0.25 mm, where the width of the V-shape is 0.20 mm. The gap between the first and second rings is 0.40 mm, the second and third ring is 0.30 mm, and the third and inverse double V-ring is 0.20 mm. All split gaps of the proposed cell are equal to 0.25 mm. The perspective view of the recommended resonator on Rogers RO4350B is shown in Fig. 1b. The full-dimensional descriptions of the MTM configuration of the unit cell are stated in Table 1.

**Table 1:** Dimensional descriptions for the suggested MTM configuration.

Parameters	Dimension (mm)	Parameters	Dimension (mm)
*W*	8	*l*_*1*_	7.5
*L*	8	*l*_*2*_	0.5
*w*_*1*_	7.5	*l*_*3*_	0.4
*w*_*2*_	0.5	*l*_*4*_	0.3
*w*_*3*_	0.4	*l*_*5*_	0.2
*w*_*4*_	0.3	*l*_*6*_	1.5
*w*_*5*_	0.4	*l*_*7*_	1.77
*w*_*6*_	0.3	*g*	0.25
*w*_*7*_	0.15	*-*	-

## Methods and techniques

**Figure 2:**
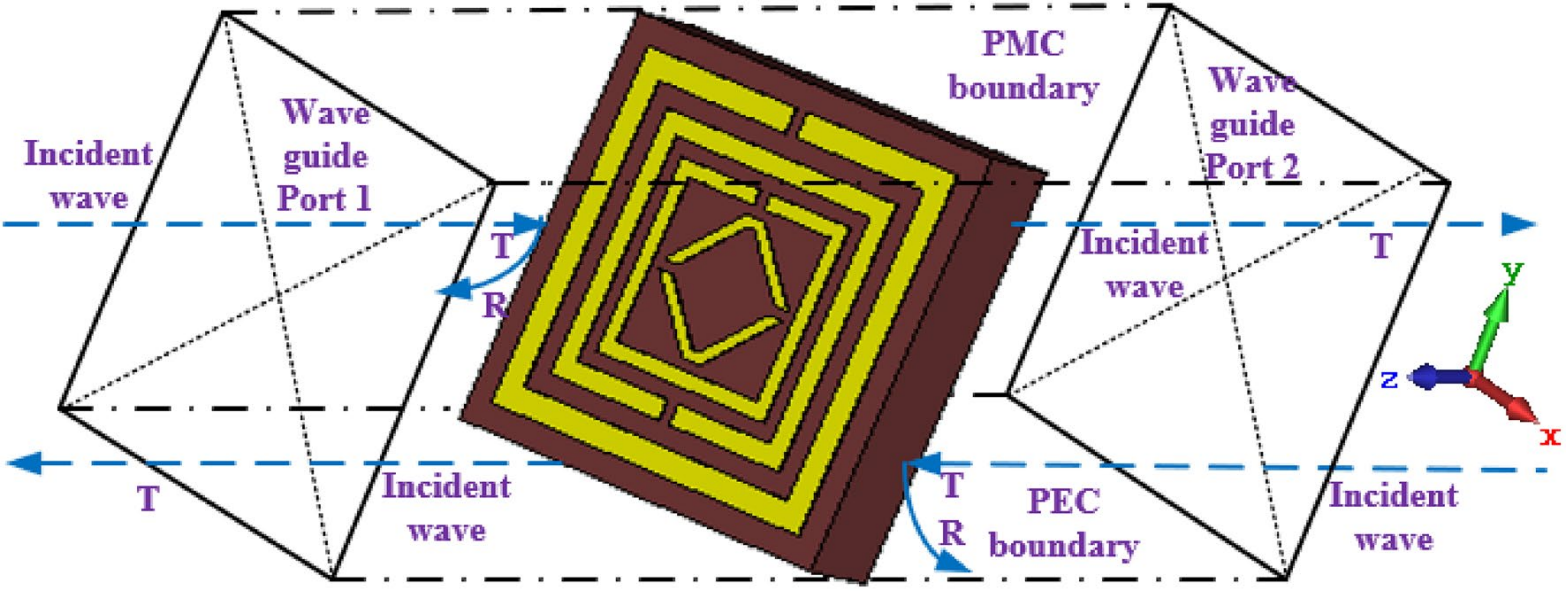
Boundary conditions are set up for the proposed structure.

Figure 2 reveals the boundary form of the suggested MTM unit cell. The suggested resonator's electromagnetic response is examined using the CST-2019 software^43^ and the finite element method. The waveguide ports are put on the negative and positive z-axes, and the V-structure is located in the middle, which is afterwards motivated in the direction of the z-axis by the EM wave. The electric field boundary condition that sets the tangential component electric field to zero is the perfect electric conductor (PEC). The magnetic equivalent of PEC boundaries is known as the perfect magnetic conductor (PMC) boundary conditions. The PEC and PMC borders and two wave ports represent the source of plane wave excitation and show that unit cells can be repeated indefinitely along the x and y directions. EM wave sources are applied from port 1 to all modes. The planes parallel to xz and yz are assigned ideal electric and ideal magnetic borders. Combining the PMC, PEC, and open space conditions of each wall aids in observing the field properties of the resonance frequencies. The suggested V-shaped cell was simulated using a frequency-domain solver with tetrahedral meshing in the frequency range of 2 to 10 GHz. From unit cell simulations in the proper frequency range, we got the complex scattering parameters S_11_ and S_21_.

The Nicolson-Ross-Weir (NRW) approach, which is one of the most widely used EM characterization techniques^44^, is used to derive the effective medium parameters, i.e., relative permittivity ($\varepsilon_{r}$), permeability ($\mu_{r}$), refractive index $(n)$, and impedance ($Z$) from the simulated S-parameter. The NRW approach begins with the application of composite terms $V_{1}$ and $V_{2}$, which add and subtract scattering properties.

$$V_{1} = S_{{11}} + S_{{21}}$$

$${V_{2}} = {S_{21}} - {S_{11}}$$

$$T = X \pm \sqrt {X^{2} - 1}$$

Here, T is the interface reflection, the $\varepsilon_{r}$, $\mu_{r}$, $n$ and $Z$ features of MTM are calculated using the equations below.

1 $$S_{11} = \frac{{\left( {1 - Z^{2} } \right)T}}{{1 - T^{2} Z^{2} }}$$

2 $$S_{21} = \frac{{\left( {1 - T^{2} } \right)Z}}{{1 - T^{2} Z^{2} }}$$

3 $$\varepsilon_{r} = \frac{2}{{jk_{0} d}}\frac{{1 + S_{11} - S_{21} }}{{1 - S_{11} + S_{21} }}$$

4 $$\mu {}_{r} = \frac{{j2S_{11} }}{{jk_{0} d}} + \mu_{0}$$

5 $$n = \frac{2}{{jk_{0} d}}\sqrt {\frac{{\left( {S_{21} - 1} \right)^{2} - S_{11}^{2} }}{{\left( {S_{21} + 1} \right)^{2} - S_{11}^{2} }}}$$

6 $$Z = \sqrt {{\raise0.7ex\hbox{${\mu_{r} }$} \!\mathord{\left/ {\vphantom {{\mu_{r} } {\varepsilon_{r} }}}\right.\kern-\nulldelimiterspace} \!\lower0.7ex\hbox{${\varepsilon_{r} }$}}}$$

Here $k_{0}$ is the wave number and $d$ is the substrate thickness.

## Design procedure of the proposed metamaterial unit cell

**Figure 3:**
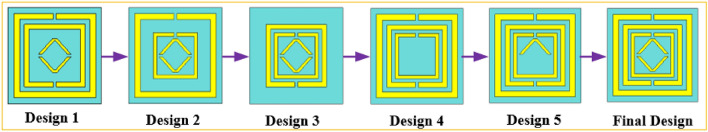
Different design layouts for selecting the final design. (CST STUDIO SUITE 2019, https://www.3ds.com/products-services/simulia/products/cst- studio-suite**)**^43^.

**Figure 4:**
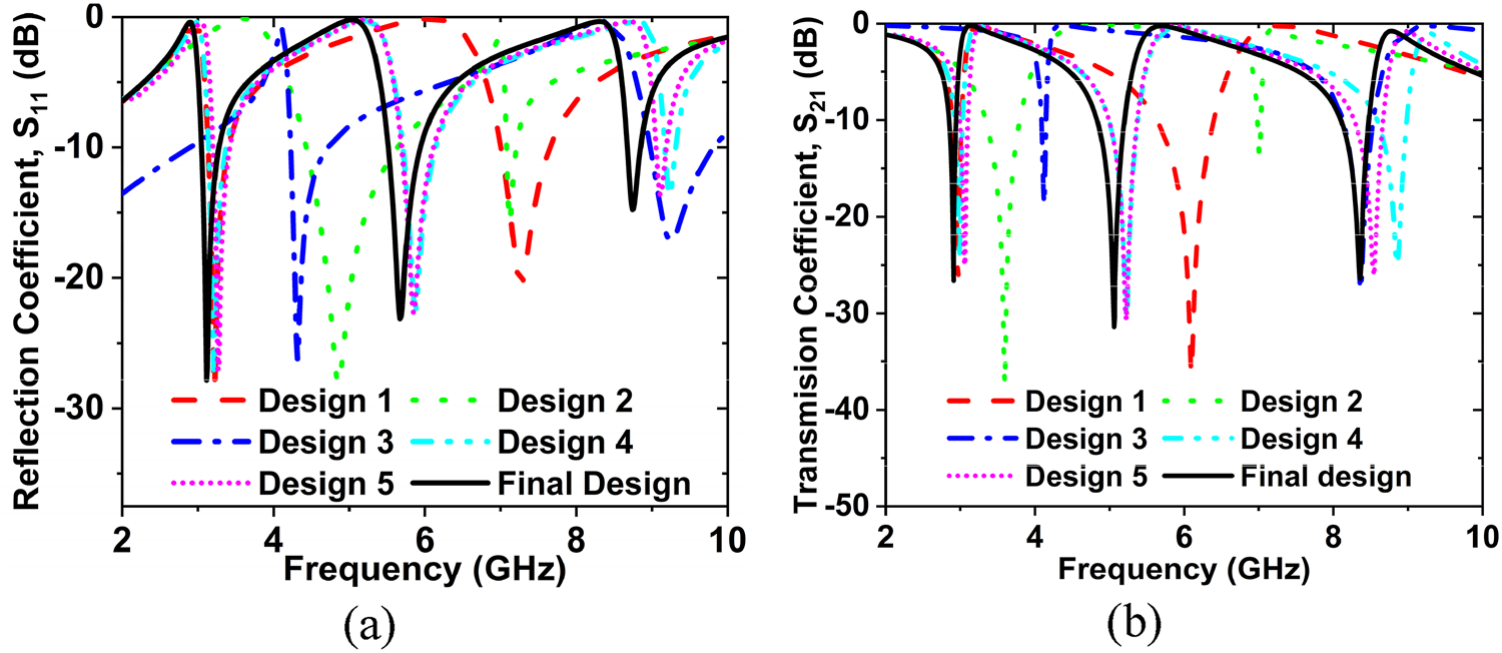
Frequency versus (**a**) S_11_ and (**b**) S_21_ for different design layouts.

We have analyzed the different designs of the MTM unit cell for selecting the intended design. Different design layouts for choosing the suggested unit cell are depicted in Fig. 3. The S_11_ and S_21_ results for the different design layouts are shown in Fig. 4(a, b). In design 1, two square split rings and inverse double V-shape resonators exist. Two resonance frequencies are obtained from it, the magnitudes of S_11_ are − 28.50, − 20.66 dB at 3.22, and 7.27 GHz, where the magnitudes of S_21_ are − 26.61, − 36.21 dB at 2.97, and 6.09 GHz, it covers S-, and C- bands. From design 2, two resonance frequencies are obtained; the magnitudes of S_11_ are − 28.13, − 15.77 dB at 4.86, and 7.12 GHz, where the magnitudes of S_21_ are − 37.39, − 13.01 dB at 3.59, and 7.01 GHz, it covers S-, and C-bands. From design 3, it is seen that the magnitudes of S_11_ are − 26.29, − 17.13 dB at 4.32, and 9.25 GHz, where the magnitudes of S_21_ are − 18.63, − 27.52 dB at 4.12, and 8.36 GHz, which also covers the S-, and C- bands. From design 4, it is seen that the magnitudes of S_11_ are − 27.07, 22.65, and − 13.65 dB at 3.21, 5.87, and 9.22 GHz, respectively, where the magnitudes of S_21_ are − 24.40, − 30.28, − 24.91 dB at 2.99, 5.22, and 8.86 GHz, and it covers S-, C-, and X- bands. The magnitudes of S_11_ are − 27.06, 22.66, − 13.66 dB at 3.27, 5.69, and 9.11 GHz, where the magnitudes of S_21_ are − 25.0, − 30.64, − 26.05 dB at 3.06, 5.22 GHz, and 8.54 GHz, which covers S-, C-, and X- bands for design 5. And the magnitudes of S_11_ are − 27.82, − 23.15, − 14.78 dB at 3.11, 5.67, and 8.74 GHz, where the magnitudes of S_21_ are − 26.86, − 31.24, − 26.16 dB at 2.86, 5, and 8.30 GHz, which covers S-, C-, and X-bands for the final design. The features of the final design are better than other designs since the desired resonance frequencies have gotten from it. Figure 4a,b demonstrates the S_11_ and S_21_ graphs for different design layouts.

## Parametric analysis

### Effect of substrate material on the MTM performance

**Figure 5:**
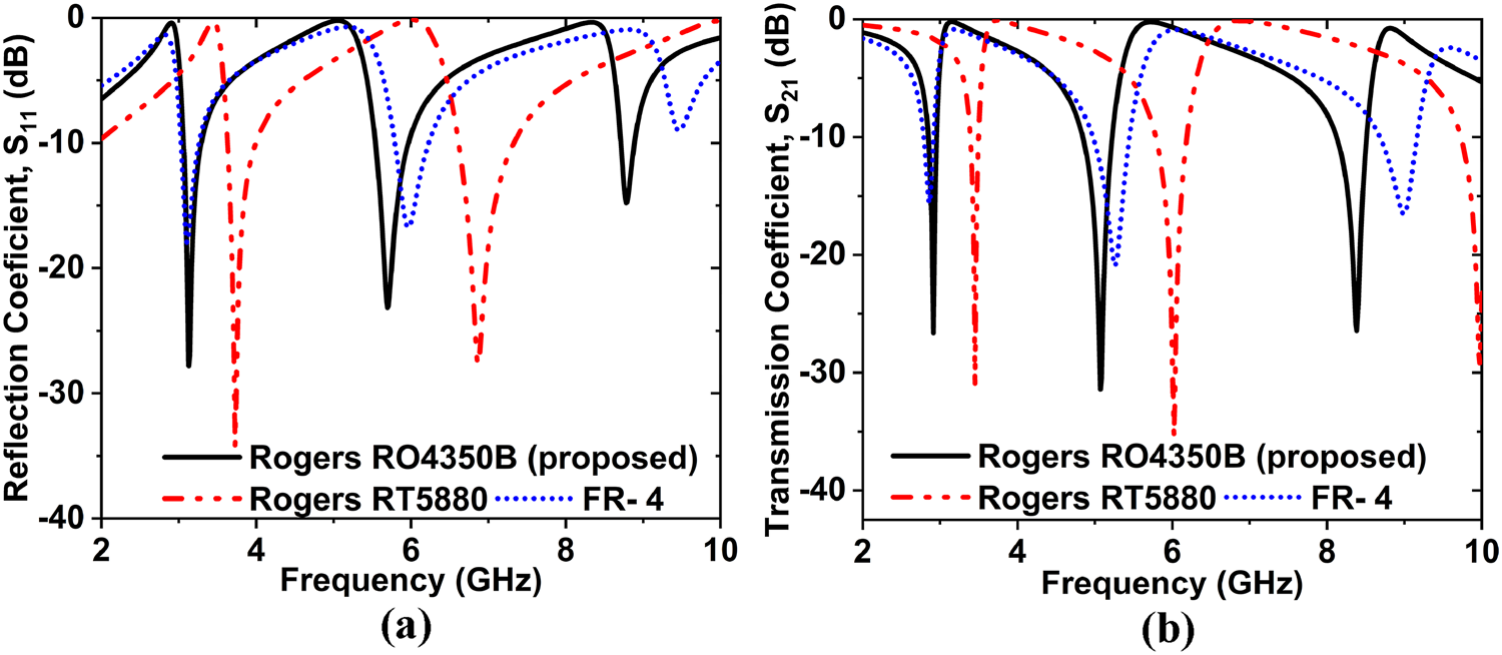
Frequency versus (**a**) S_11_ and (**b**) S_21_ curves for the different substrate materials.

Several types of substrate material have been used to justify the forms of the suggested MTM unit cell. Substrate materials are Rogers RO4350B, RT5880, and FR-4. Dielectric constant (DK), loss tangent (LT), and thickness are 3.66, 0.037, and 1.524 mm for Rogers RO4350B, 2.2, 0.009, 1.575 mm for Rogers RT5880 and 4.3, 0.025, 1.5 mm for FR-4, respectively. When Rogers RO4350B was used as a substrate, the magnitudes of S_11_ were − 27.81 dB at 3.13 GHz, − 23.15, − 14.78 dB at 5.69, 8.77 GHz and the amplitudes of S_21_ were − 26.86, − 31.24, − 26.16 dB, at 2.86, 5, 8.30 GHz, which covers three bands S-, C-, and X-. When RT5880 was used, the magnitudes of S_11_ were − 34.23 dB at 3.73 GHz, − 27.64 dB at 6.87 GHz, and the values of S_21_ are − 31.32, − 35.39 dB at 3.45, 6.02 GHz, which covers S-, and C- bands. When FR-4 was used as a substrate, the magnitudes of S_11_ were − 17.98 dB at 3.10 GHz, − 16.75, − 9.03 dB at 5.96, 9.46 GHz and the values of S_21_ were − 15.60, − 20.92, and − 16.55 dB at 2.86, 5.26, and 9 GHz, respectively, it covers three bands. Figure 5a,b signifies the frequency versus S_11_ and S_21_ curves for different substrate materials. Since the permittivity value of the material is directly proportional to the capacitance (C), and C is inversely proportional to the resonance frequency. Hence the resonance frequency differs for Rogers RO4350B, RT5880, and FR-4 due to the different permittivity values, indicating that the MTM's performance is dependent on the substrate material. The resonance frequency of the MTM is a shift to the higher value for the lower value of substrate permittivity and a shift to the lower value for the higher value of substrate permittivity. Since the features such as covering band, EMR, desired frequency and magnitude of S_11_ and S_21_ of the Rogers RO4350B are better than others, so Rogers RO4350B is finally selected as a substrate material.

### Effect of variations of split gap

**Figure 6:**
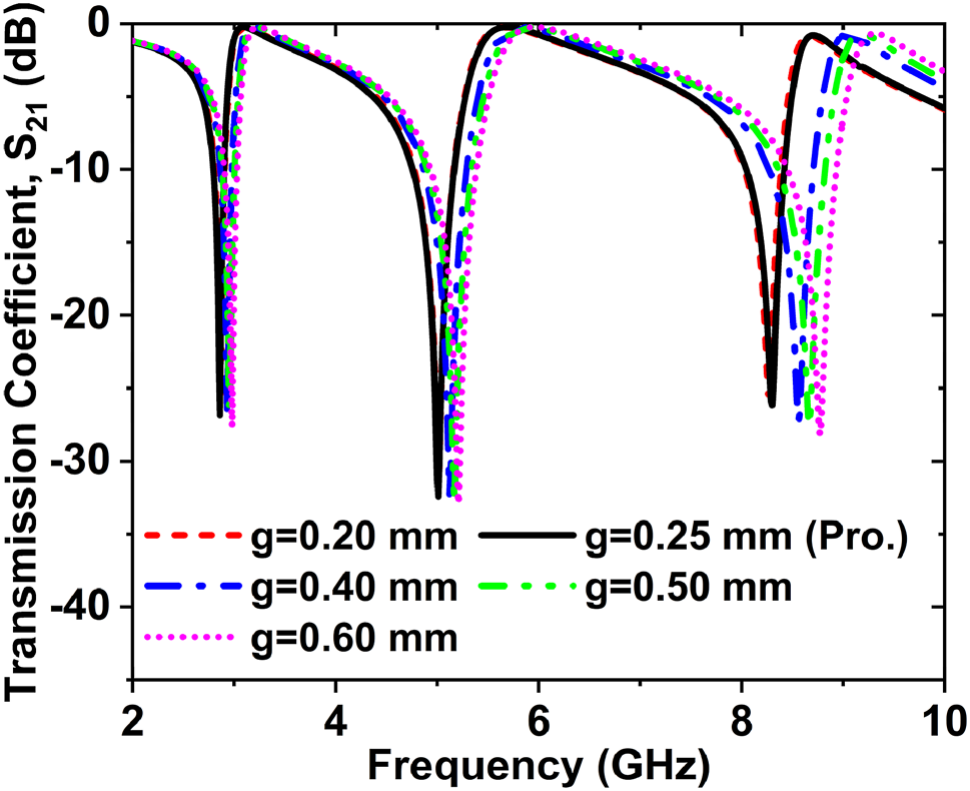
Effect on S_21_ for changing the split gap.

The impact of split gap variations on transmission coefficient (S_21_) is exhibited in Fig. 6. In this analysis, five different split gap such as 0.2, 0.35, 0.4, 0.5, and 0.6 mm has been used to see the best performance of the MTM cell. The values of S_21_ are − 25.72, − 31.81, − 25.75 dB at 2.86, 4.99, and 8.27 GHz for 0.20 mm gap. The values of S_21_ for split gap 0.25 mm are − 26.86, − 31.24, − 26.16 dB at 2.86, 5 and 8.30 GHz, respectively. When the structure’s split gap is 0.40 mm, the magnitudes of S_21_ are − 26.75, − 32.26, − 27.24 dB at 2.93, 5.12 and 8.56 GHz, respectively. If the cell’s split gap is 0.50 mm, then the amplitudes of S_21_ are − 26.26, − 32.62, − 27.59 dB at 2.95, 5.17, and 8.67 GHz, respectively. The magnitudes of S_21_ are − 27.62, − 32.92, and − 28.18 dB at 2.98, 5.21, and 8.77 GHz for a 0.60 mm gap. The resonance frequency increases with increasing the split gap. We can see that the performance of the MTM unit cell for the gap of 0.50 mm is better than the other split gap. Finally, a 0.50 mm gap was selected for the proposed structure.

### Effect of several types of conductor on transmission coefficient

**Figure 7:**
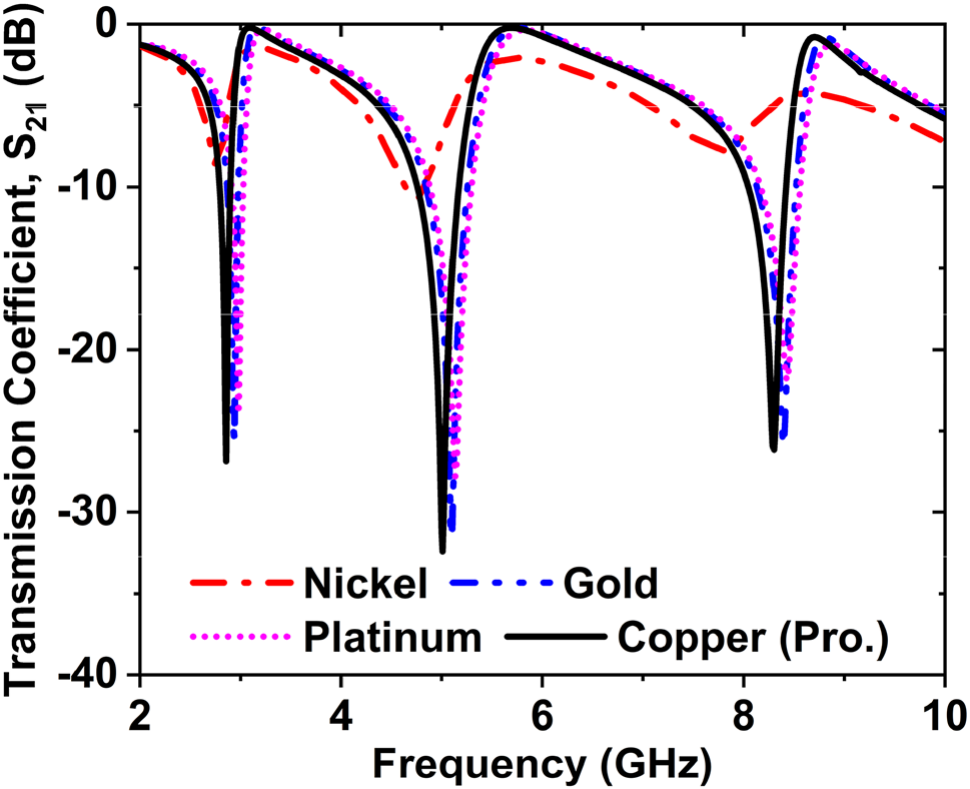
Effect on S_21_ for changing the conductor materials.

Various conductor effects on the S_21_ is depicted in Fig. 7. We have used four different metal conductors such as nickel, gold, platinum, and copper. When the nickel is used as a patch on the substrate, the magnitudes of S_21_ are − 8.81, − 10.69, and − 7.78 dB at 2.77, 4.74, and 7.76 GHz, respectively. The magnitudes of S_21_ are − 25.53, − 31.53, and − 25.89 dB at 2.94, 5.10, and 8.39 GHz for gold. If the platinum is used as a patch, then the magnitudes of S_21_ are − 23.60, − 27.81, − 21.89 dB at 2.98, 5.13, and 8.43 GHz. When we used copper as a patch on the substrate, the magnitudes of S_21_ were − 26.86, − 31.24, and − 26.16 dB at 2.86, 5, and 8.30 GHz. Since the performance of the copper as a patch is better than others, finally, copper is selected as a patch.

### Effect of structure dimension variations

**Figure 8:**
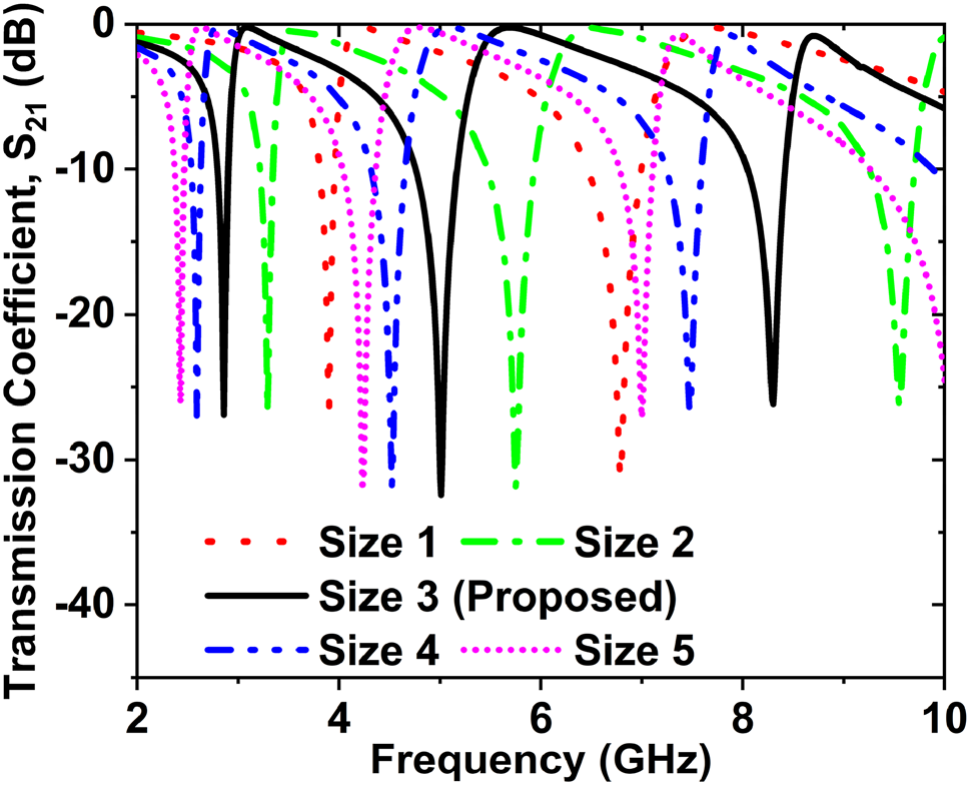
Effect on S_21_ for changing the unit cell size.

Figure 8 shows the S_21_ curve for different sizes of the inverse double V-shaped unit cell. We have taken five different sizes of the MTM structure for delivering the best performance. The different sizes of the MTM cell are 6 $\times$ 6, 7 $\times$ 7, 8 $\times$ 8, 9 $\times$ 9, and 10 $\times$ 10 mm^2^. If the unit cell size is 6 $\times$ 6 mm^2^, the S_21_ are 3.90, 6.79 GHz with amplitude − 26.35 and 31.31 dB, respectively. The EMR 12.82 and including bands are S-, and C-. The S_21_ resonance frequencies are 3.29, 5.75, and 9.55 GHz, with magnitudes − 26.72, − 31.83, and − 26.25 dB respectively; EMR is 13.03 and including bands are S-, C-,and X- for the size of 7 $\times$ 7. When the size is 8 $\times$ 8 mm^2^, including bands are S-, C-, X-, magnitudes of S_21_ are − 26.86, − 31.24, and − 26.16 dB at 2.86, 5, and 8.30 GHz and the EMR is 13.11. The EMR is 12.87, covering bands S-, C-, magnitudes of S_21_ are − 27.03, − 31.81, and − 26.61 dB at 2.59, 4.52, and 7.48 GHz for the unit cell size 9 $\times$ 9. The magnitudes of S_21_ are − 26.13, − 31.73, and − 27.09 dB at 2.43, 4.23, and 7.01 GHz; EMR is 12.35, covering bands S-, and C-, for the unit cell size 10 $\times$ 10 mm^2^. After analysing the cell size, the number of resonance frequency, covering bands, desired frequency and high EMR value 13.11 were found for the 8 $\times$ 8 mm^2^ size, which is better than the others. So the structure size 8 $\times$ 8 mm^2^ has been fixed for this MTM structure.

### Effect of different thicknesses of the substrate

**Figure 9:**
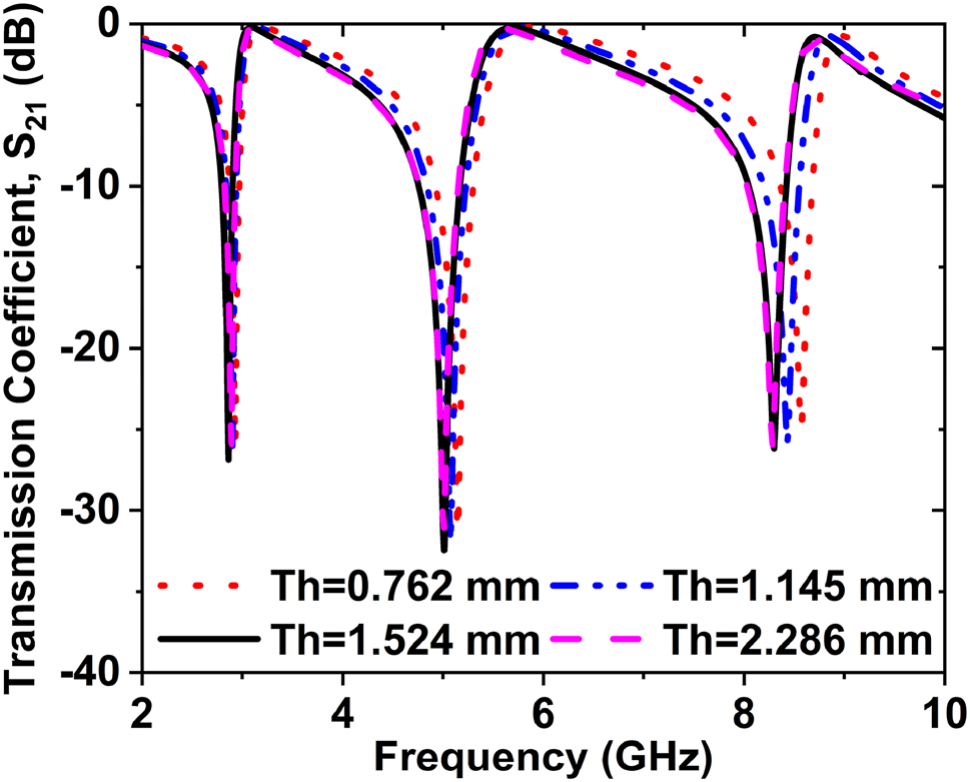
Effect on S_21_ for changing the thickness of the substrate.

Figure 9 symbolizes the impact of different thicknesses of the substrate on the transmission result (S_21_). We have taken four (4) different thicknesses of the substrate to investigate the response of the S_21_. Four (4) thicknesses are 0.762, 1.145, 1.524, and 2.286 mm. The amplitudes of S_21_ are − 27.97, − 31.29, and − 24.80 dB at 2.93, 5.15, and 8.57 GHz, respectively for 0.762 mm. The S_21_ amplitudes are − 26.22, − 31.97, and − 25.85 dB at 2.90, 5.07, and 8.43 GHz, respectively for the 1.145 mm. If the substrate thickness is 1.524 mm, then the amplitudes of S_21_ are − 26.86, − 31.24, and − 26.16 dB at 2.86, 5, and 8.30 GHz, respectively. When the substrate thickness 2.286 mm was taken, the magnitudes of S_21_ were − 26.09, − 31.35, and − 26.08 dB at 2.89, 5.01, and 8.31 GHz, respectively. In Fig. 9, the resonance frequency decreases with the increased thickness. Since the magnitude and desired resonance frequency for the substrate thickness of 1.524 mm is better than others, it is selected for the thickness of the substrate.

### Analysis of the axis of waveguide port

**Figure 10:**
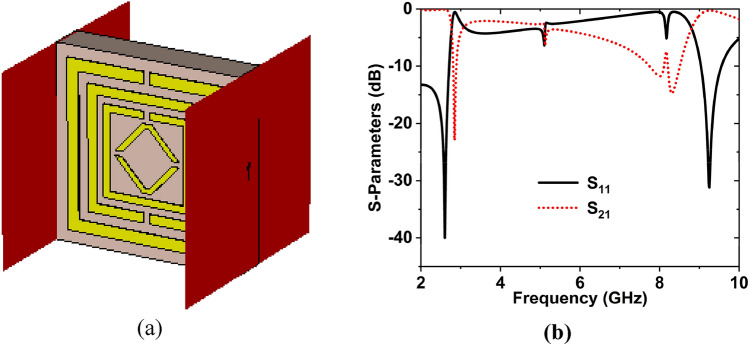
Wave propagation in the x-axis: (**a**) Simulation structure (**b**) S-parameters. (CST STUDIO SUITE 2019, https://www.3ds.com/products-services/simulia/products/cst- studio-suite**)**^43^.

**Figure 11:**
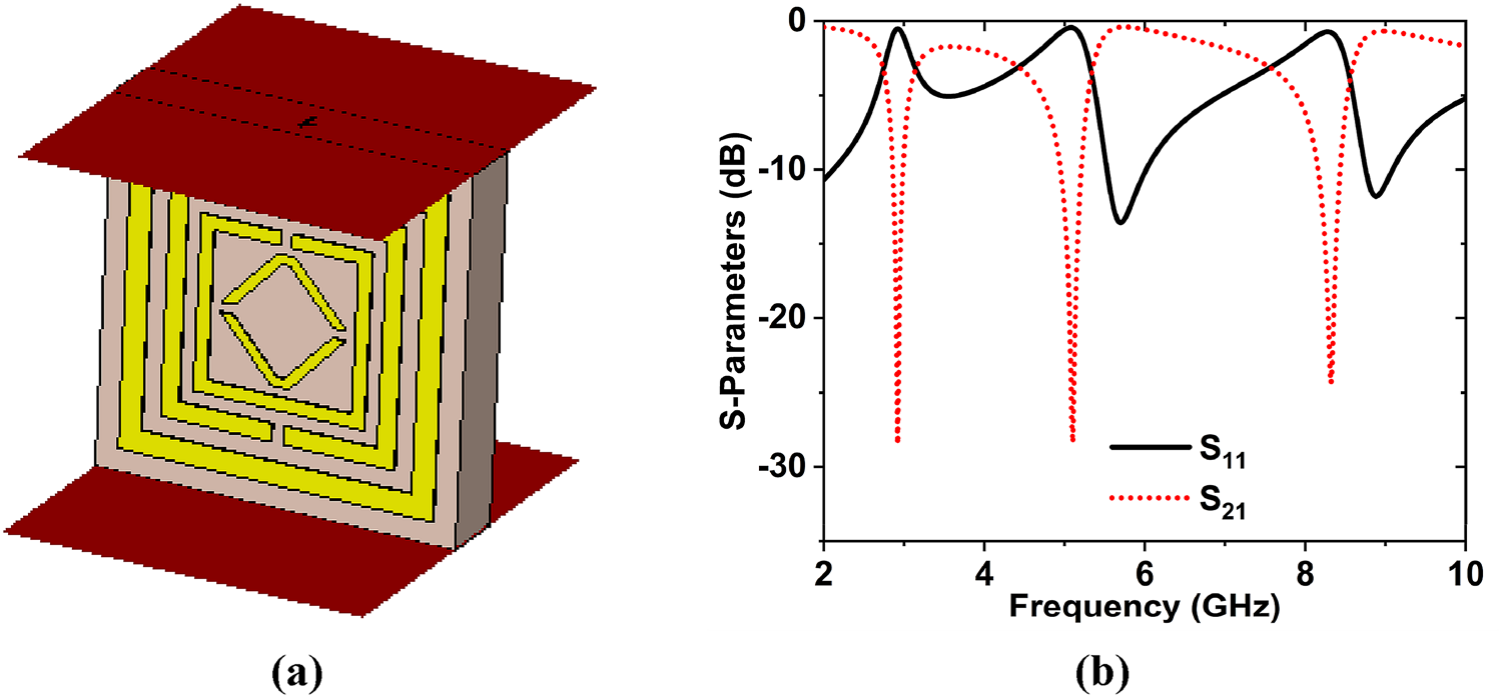
Wave propagation in the y-axis: (**a**) Simulation arrangement (**b**) S-parameters.

The MTM structure was also analyzed in the x- and y-axes to see how the results changed. Figure 10a,b depicts the simulation arrangement and scattering parameters in the x-axis wave propagation. Figure 10b shows that the reflection coefficient magnitudes are − 39.97, − 31.19 dB at 2.60, and 9.25 GHz, whereas the transmission coefficient magnitudes are − 22.91, − 14.74 dB at 2.84, and 8.31 GHz, respectively. When a wave propagates along the y-axis**,** the S_11_ magnitude values are − 13.58, − 11.80 dB at 5.70, and 8.88 GHz, whereas the transmission magnitude values are − 28.45, − 28.35, and − 24.34 dB at 2.92, 5.10, and 8.32 GHz, respectively. These two investigations revealed that neither of the wave propagations exhibits the appropriate resonance frequencies. In the meantime, superior results were obtained in the z-axis. As a result, the lack of resonance frequencies and distinct properties, x- and y-axes wave propagations were rejected.Fig. 11a represents the simulation configuration, and Fig. 11b depicts the S-parameters for the y-axis wave propagation in the suggested structure.

## MTM array analysis

**Figure 12:**
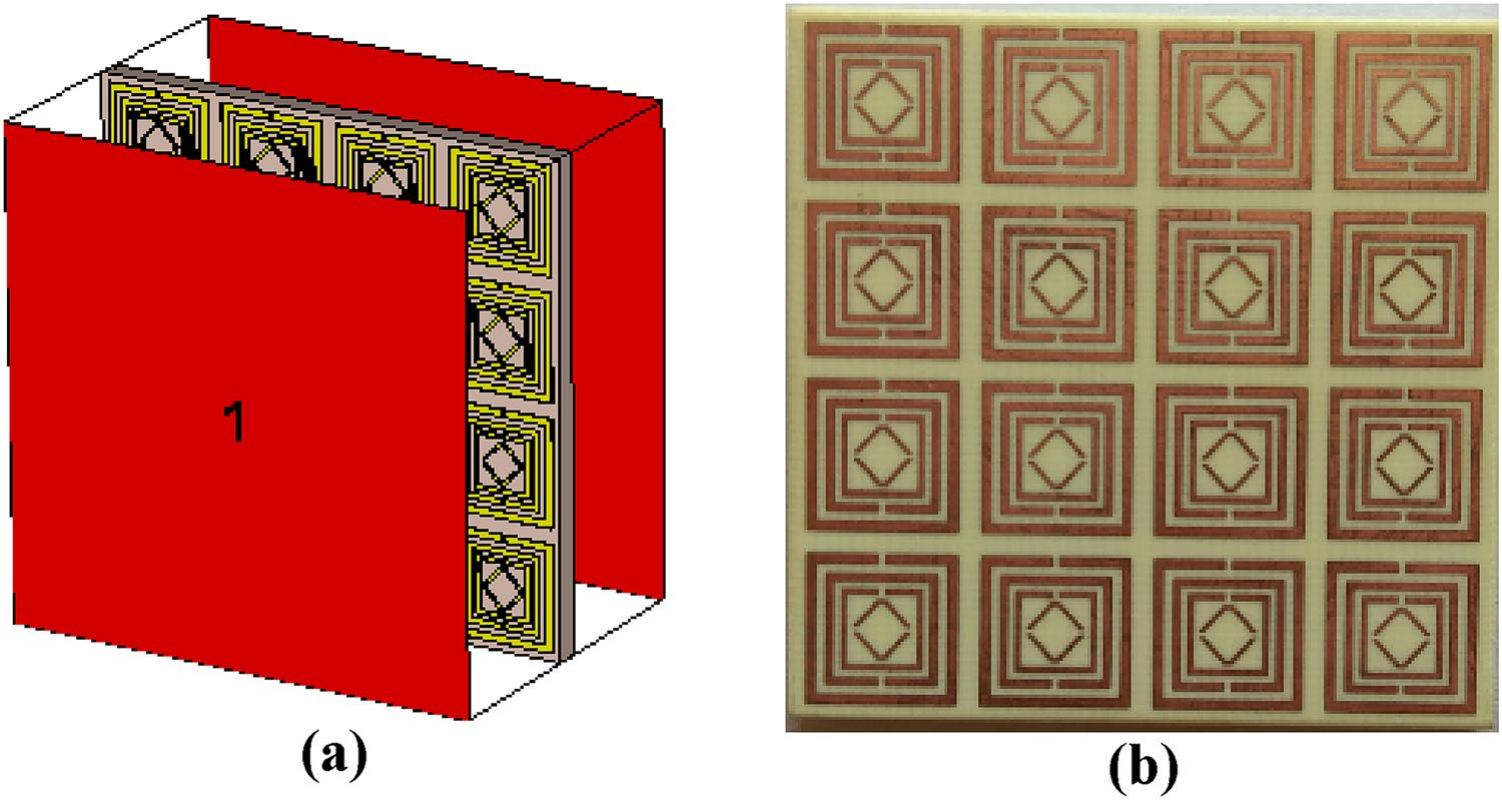
The 4 $\times$ 4 array (**a**) simulated arrangement (**b**) fabricated design.

In general, a single unit cell MTM cannot function on its own; rather, it requires an array of unit cells to demonstrate acceptable exotic EM properties. On the other hand, an MTM containing electrically conductive parts is an array with sufficient capacitive and inductive properties. Figure 12 shows the simulation setup and fabricated design of a 4 $\times$ 4 array of V-shaped MTM unit cells.

**Figure 13:**
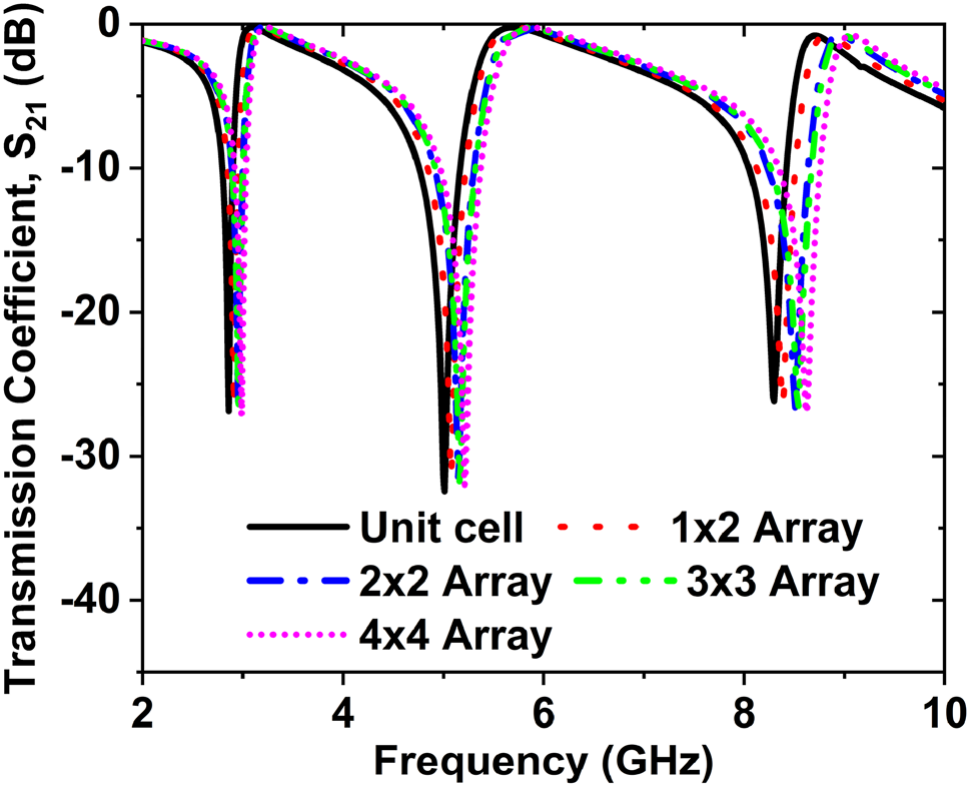
Effect on S_21_ for a different array of the V-shaped cell.

As shown in Fig. 13, several array arrangements were chosen for this parametric study, including 1 × 2, 2 × 2, 3 × 3, and 4 × 4 arrays. All four array cells delivered multi resonance frequencies in the S-, C-, and X-bands; the magnitude values and frequency deviated slightly from unit cell results. Table 2 represents the various features of the array.

**Table 2:** The transmission resonance frequency and its magnitude for different arrays.

Configuration	Resonance frequency (GHz)	Magnitude (dB)	EMR
Unit cell	2.86, 5, 8.3	− 26.86, − 31.24, − 26.16	13.11
1 $\times$ 2 array	2.91, 5.07, 8.38	− 26.62, − 31.41, − 26.43	12.89
2 $\times$ 2 array	2.96, 5.15, 8.51	− 26.89, − 31.72, − 26.69	12.67
3 $\times$ 3 array	2.96, 5.16, 8.54	− 27.02, 31.87, 26.83	12.96
4 $\times$ 4 array	2.99, 5.21, 8.62	− 27.15, − 32.03, − 26.96	12.54

## Equivalent circuit analysis

**Figure 14:**
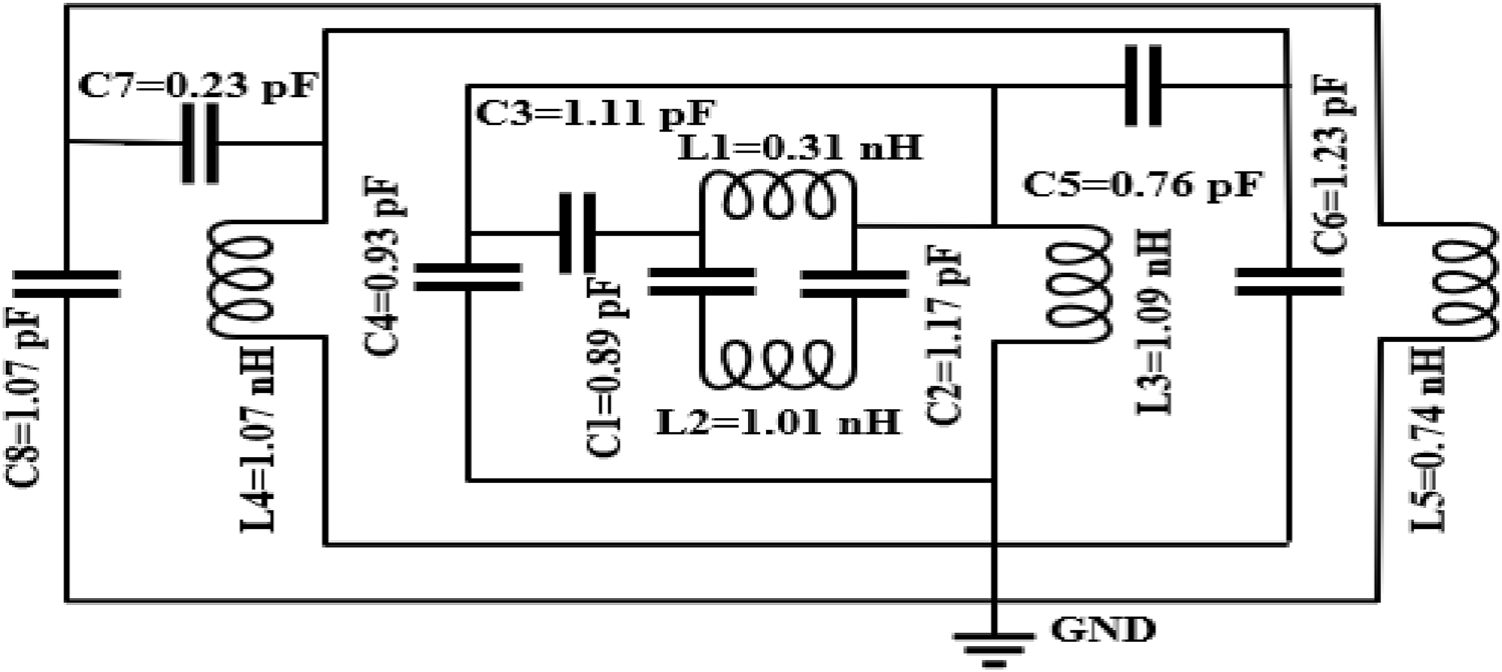
Equivalent circuit of the proposed structure. (Path Wave Advance Design System (ADS) https://www.keysight.com/sg/en/lib/resources/software-releases/pathwave-ads-2019.html)^45^.

The equivalent circuit of the CST and HFSS simulated unit cell has been made by the Advanced Design System (ADS-2019) software^45^ shown in Fig. 14. In this designed equivalent circuit, inductances (*L*)and capacitances (*C*) are used to create the resonance point of transmission response (S_21)_. The metal strip produces inductances, and capacitances are produced by the split gap of the designed unit cell^46^. The *LC* circuit is constructed the transmission resonance frequency to validate the CST and HFSS simulated transmission response of the MTM structure. The inverse double V-ring forms the inductances and capacitances *L1, L2, C1*, and C2. *L3* and *C4* are formed by the second square ring, where *C3* is the coupling capacitance created by the gap between inverse double V and the second square ring. The third square ring forms *L4 and C6*; here, *C5* is the coupling capacitance formed by the gap between the second and third square rings. *L5* and *C8* are created by the fourth square ring, where *C7* is the coupling capacitance produced by the gap between third and fourth square rings. Individual inductance and capacitances are *L1, L2, L3, L4, L5,* and *C1, C2, C3, C4, C5, C6, C7*, and *C8*. The transmission resonance frequency *(f*_*r*_*)* can be stated by the equation.

7 $$f_{r} = \frac{1}{{2\pi \sqrt {LC} }}$$

**Figure 15:**
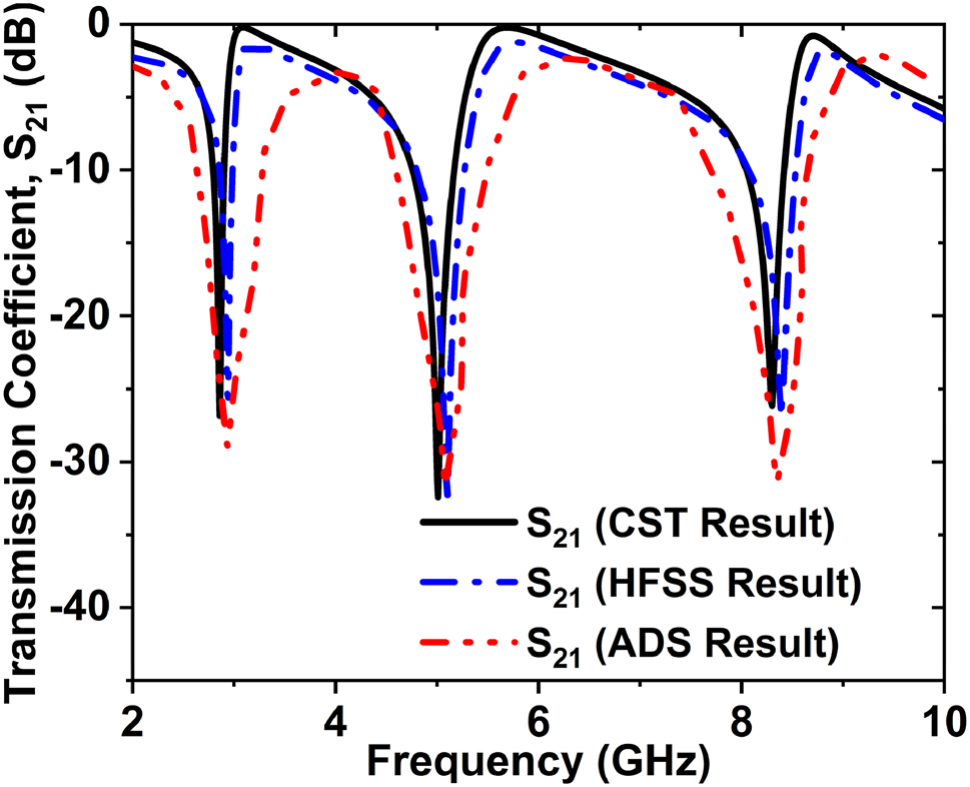
Transmission coefficient (S_21_) graph for CST, HFSS, and ADS simulation.

Here, *C* is the capacitance that is created by the split gap and can be expressed by the resulting equation

8 $$C = \varepsilon_{0} \varepsilon_{r} \frac{A}{d}(F)$$

where $\varepsilon_{0}$=$8.854\times {10}^{-12}$ F/m, $\varepsilon_{r}$ = relative permittivity, $A$ and $d$ are the area and distance of the split, respectively. And *L* is the inductance generated by the metal strip, which the equation can present

9 $$L\left( {nH} \right) = 2 \times 10^{ - 4} \left[ {\ln \left( {\frac{l}{w + t}} \right) + 1.193 + 0.02235\left( {\frac{w + t}{l}} \right)} \right]K_{g}$$

where $K_{g}$ denote the adjustment factor, $K_{g} = 0.57 - 0.145\ln \frac{{w^{\prime}}}{{h^{\prime}}}$ here ${w}^{^{\prime}}$ and ${h}^{^{\prime}}$ are the width and thickness of the substrate. Also,$t$, $l$, and $w$ are the microstrip line thickness, length, and width correspondingly. To attain overall inductance, it's vital to accept both internal and external inductance in this equivalent circuit; the values of inductance and capacitance components are obtained by using the ADS software considering the desired response of S_21_. By tuning the component values in ADS, the values are so chosen that it provides similar resonances of S_21_ obtained from the CST and HFSS. To adjust the first transmission resonance frequency to 2.86 GHz, we tuned the inductor *L3* and capacitor *C4*. When the second resonance 5 GHz is fixed, the inductor *L4* and capacitors *C6* has been tweaked. The inductor *L5* and capacitor *C8* are tuned to adjust the third resonance frequency 8.30 GHz. Finally, the CST, HFSS and ADS simulated transmission coefficient is presented in Fig. 15, where these three results are almost the same.

## Surface current, E-field, and H-field analysis

**Figure 16:**
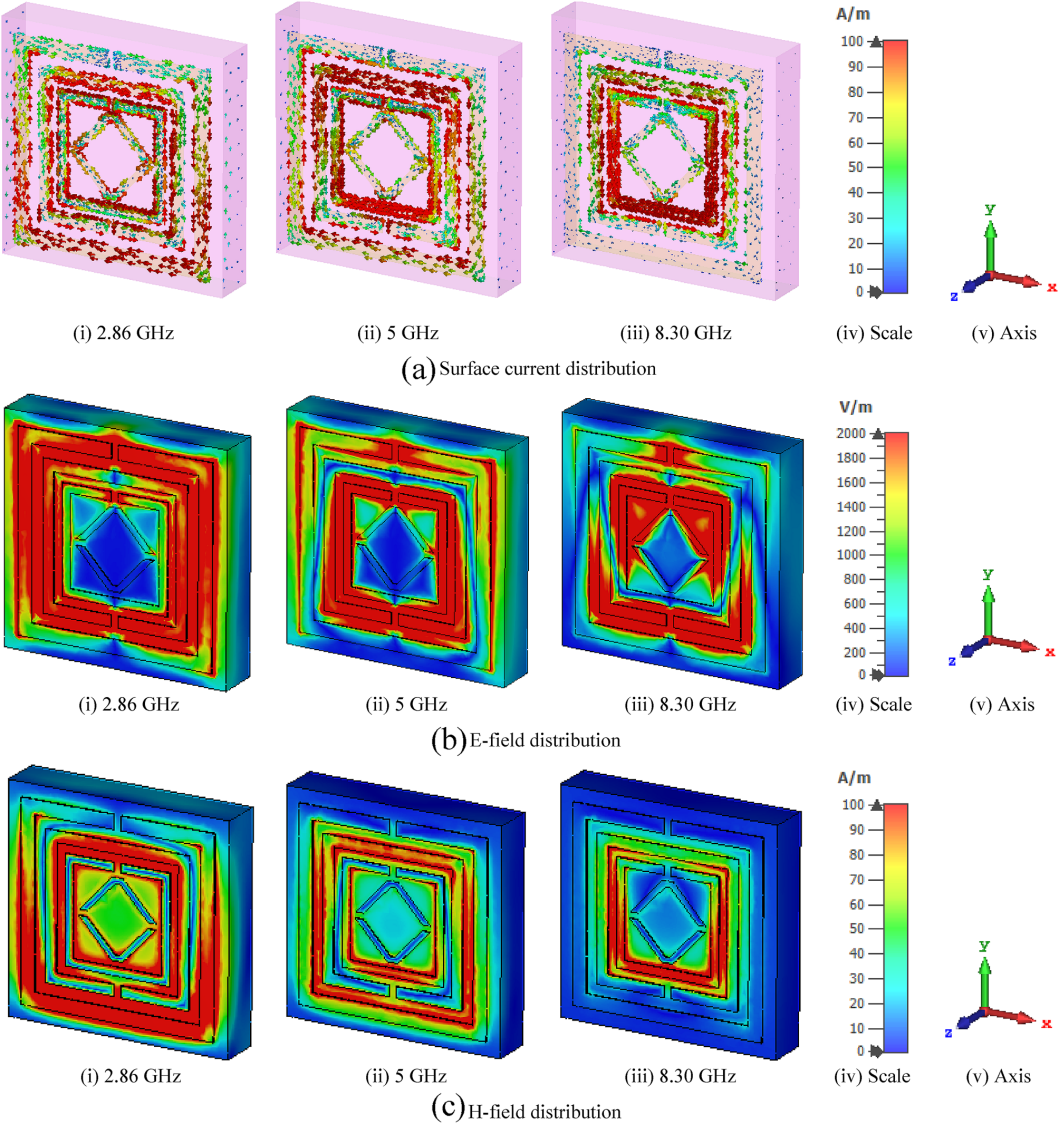
Analysis of (**a**) Surface current (**b**) E-field and (**c**) H-field for the designed structure. (CST STUDIO SUITE 2019, https://www.3ds.com/products-services/simulia/products/cst- studio-suite**)**^43^.

The surface current of the produced MTM unit cell is described for different transmission resonance frequencies. Figure 16a shows the surface current pattern of three distinct resonances that occurred at frequencies of 2.86, 5, and 8.30 GHz, respectively. The current is distributed on the surface of the patch. For the first resonance frequency, 2.85 GHz, current follows equally in every metal ring which is mentioned by the red colour. The intensity of the surface current is high for the upper portion of the second ring and the lower portion of the third ring, as depicted in 5 GHz. The outer ring and opposite double V-shape resonator observed the lower intensity of the current. The inner ring feels the high intensity of the current, and the middle ring shows a moderate level of current for 8.30 GHz resonance frequency. The outer ring and V-ring noticed the minimum intensity of the current. The E-field and H-field can be described by the following equations^47^.

10 $$\nabla \times H = J + \frac{\partial D}{{\partial t}}$$

11 $$\nabla \times E = - \frac{\partial B}{{\partial t}}\;{\text{where}}\;\nabla = \left[ {\frac{\partial }{\partial x},\frac{\partial }{\partial y},\frac{\partial }{\partial z}} \right]$$

Two more equations can be used to determine the electromagnetic field's interaction with materials^48^.

12 $$D(t) = \varepsilon (t) \times E(t)$$

13 $$B(t) = \mu (t) \times H(t)$$

The E-field and H-field distributions have been taken in absolute components with instantaneous plot attributes and CST simulation outside orientation at different frequencies. So, the z-component of E-field and H-field distributions (Ez and Hz) of the absolute of E-field and H-field distributions (|E| and |H|). Figure 16b shows the E-field of the aforementioned MTM unit cell at three distinct resonance frequencies. These frequencies are 2.86, 5 and 8.30 GHz. The E-field is almost equally noticeable around the different rings at the 2.86 GHz resonance frequency. The distribution of the E-field is more concentrated in the inner ring and slightly concentrated on the middle and outer ring of the cell for 5 GHz transmission resonance frequency. The lower portion of the middle ring and upper portion of the inner ring noticed a high charge, but the outer ring and double V-shape noticed a very small charge for 8.3 GHz resonance frequency. The break or gap in the resonator ring creates a capacitor, which accumulates electric charge and produces additional E-fields; the E-field strength is significant at certain points. At a reserver, the H-field around a wire follows the equation $B = {{\mu I} \mathord{\left/ {\vphantom {{\mu I} {2\pi r}}} \right. \kern-\nulldelimiterspace} {2\pi r}}$; here $\mu$ is the permeability of the free space. For all resonance frequencies, the H-field and E-field show nearly opposing excitation. The magnetic field is formed by the movement of electrical charges in general. As a result, maximal charge mobility boosts magnetic field strength, as evidenced by the magnetic field (also known as the H-field). Figure 16c represents the H-field distribution for three individual resonance frequencies. When a transverse EM wave propagates through a metamaterial for a specific frequency spectrum, an artificial magnetic dipole moment is introduced in a split ring resonator. The three different resonance frequencies are 2.86 GHz, 5, and 8.30 GHz, and for each resonance frequency, the H-field behaviour has been shown. The h-field are strong in the lower position of the outer and middle ring and surroundings of the inner ring. The opposite double V shape resonator noticed moderate field intensity for 2.86 GHz resonance frequency. It is also noticeable that the field intensity is moderate in the middle and inner ring, but it is low in the outer and V shape resonator for the resonance frequency 5 GHz. The intensity of the field is much visible in the most inner ring, but the so slight intensity is observed in the most outer, middle and V shape resonator for the resonance frequency 8.30 GHz.

## Results and discussion

**Figure 17:**
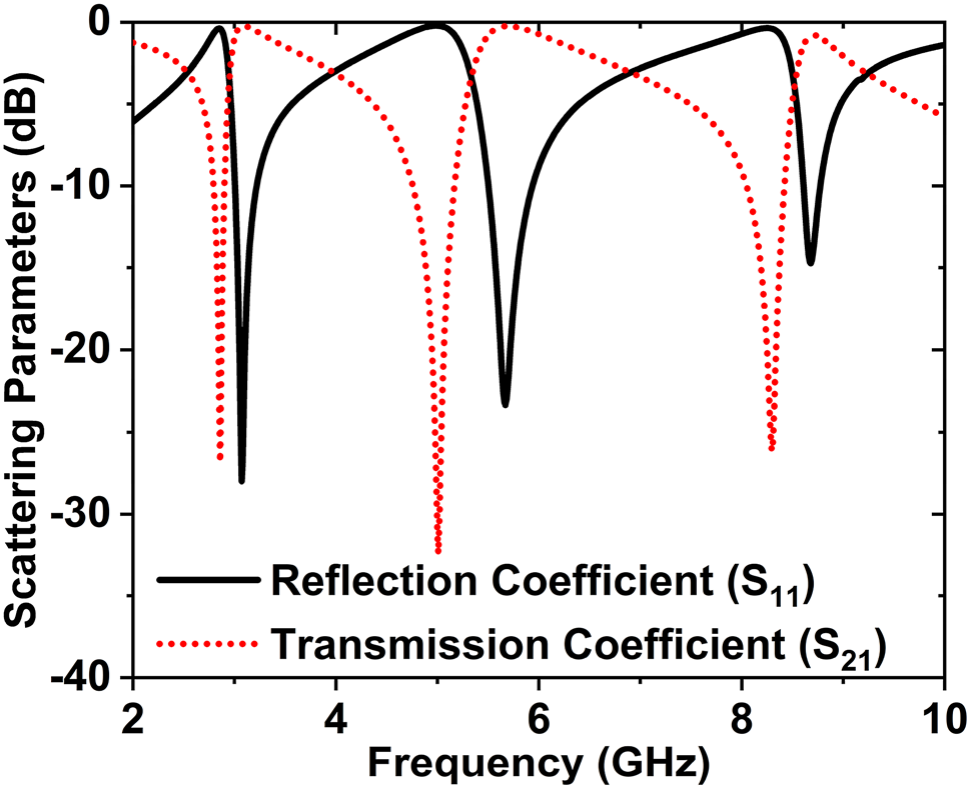
S-parameter graph of the suggested V-structure.

The S-parameter of the recommended unit cell is shown in Fig. 17. The reflection and transmission factor has three resonance frequencies. Three resonance frequencies of S_11_ are 3.07, 5.67, and 8.68 GHz with magnitudes of − 27.99, − 23.35, and − 14.72 dB, respectively. The − 10 dB bandwidth of S_11_ is 3.01 to 3.22, 5.51 to 5.94, and 8.61 to 8.77 GHz. Similarly, the three resonance frequencies of S_21_ are 2.86, 5, and 8.30 GHz with magnitudes of − 26.86, − 32.42, and –26.16 dB, respectively. The − 10 dB bandwidth of S_21_ is 2.80 to 2.91, 4.76 to 5.17, and 8.05 to 8.42 GHz.

**Figure 18:**
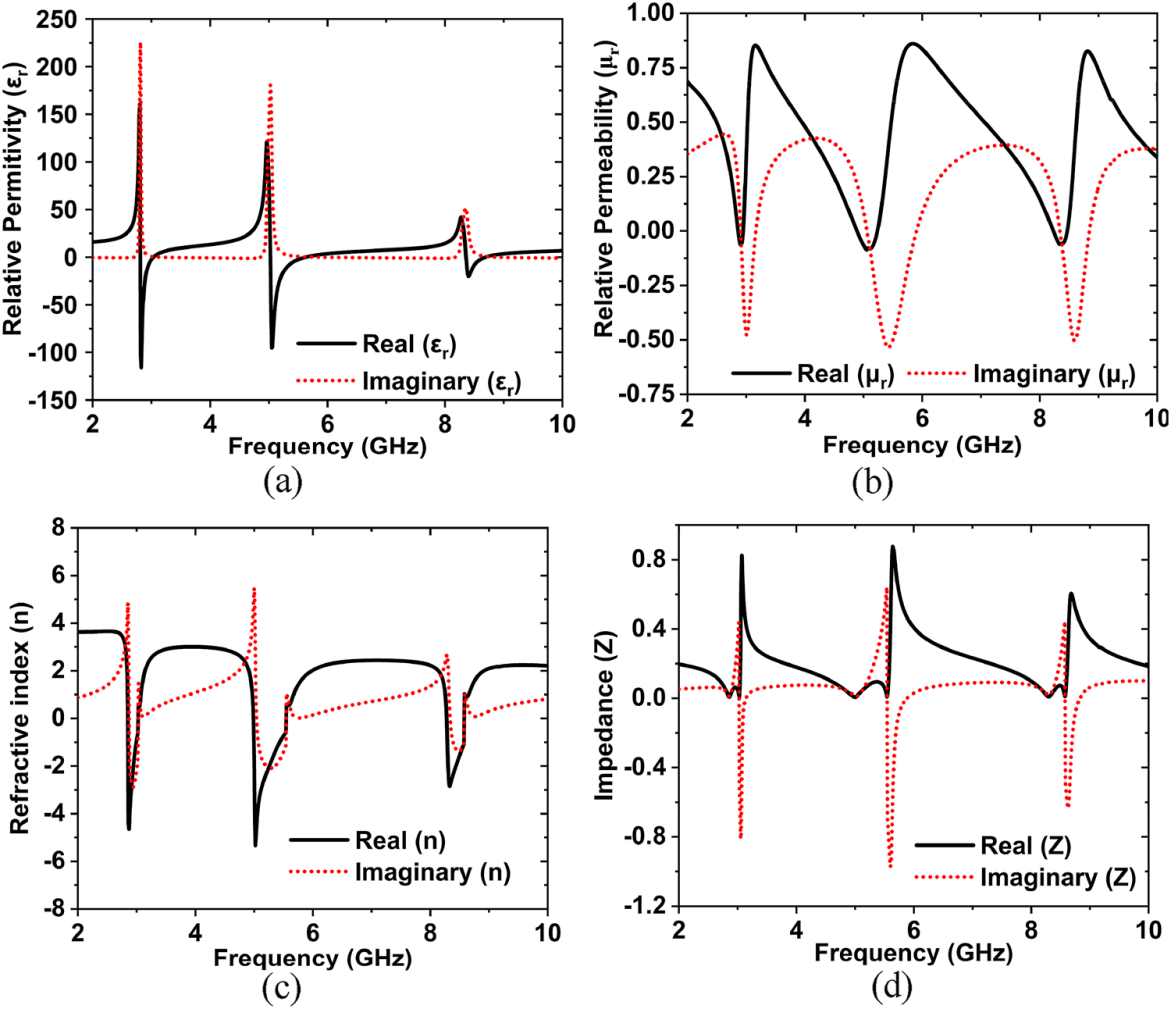
Frequency versus: (**a**) Relative permittivity (**b**) Relative permeability(**c**) Refractive index and (**d**) Impedance.

The effective medium parameters ${\varepsilon }_{r}$, ${\mu }_{r}$, $n$, and Z of the inverse double V-structure is derived from the scattering parameters using the NRW approach, which was addressed by Eqs. (3–6). The NRW based MATLAB code is used for obtaining the features of ${\varepsilon }_{r}$, ${\mu }_{r}$, $n$, and Z^49^. The information extracted from these qualities using the NRW approach can be compared to CST findings. Figures 18a–d shows the extracted real and imaginary parts of the ${\varepsilon }_{r}$, ${\mu }_{r}, n$, and Z, from the MATLAB program. It is visible that the designed MTM unit cell shows the epsilon and mu negative, i.e., double negative. The frequency range of epsilon negative is 2.82 to 3.03, 5.04 to 5.62, and 8.36 to 8.68 GHz. From Fig. 18b, we can see that the negative region of relative permeability (real part) is 2.81 to 2.89, 4.89 to 5.23, and 8.24 to 8.46 GHz and the negative peaks are − 0.064 dB at 2.91 GHz, − 0.081 dB at 5.1 GHz, and − 0.061 dB at 8.34 GHz. Hence the double negative regions of the inverse double V-shaped MTM unit cell are 2.82 to 2.89, 5.04 to 5.23, 8.36 to 8.46 GHz. Refractive index ($n$) is also negative; these ranges are 2.84 to 2.99, 4.97 to 5.52, and 8.26 to 8.54 GHz, as presented in Fig. 18c. The impedance values are as well reported in this study, as seen in Fig. 18d. The suggested inverse double V structure had impedance values of 0.23, 0.12, 0.29, and 0.27 at 5.89, 7.70, 10.37, and 12.58 GHz, respectively. In the meantime, the real impedance values were kept positive across the whole simulated frequency range. To validate the simulation result, unit cell and array prototypes have been fabricated and taken the experimental results.

**Figure 19:**
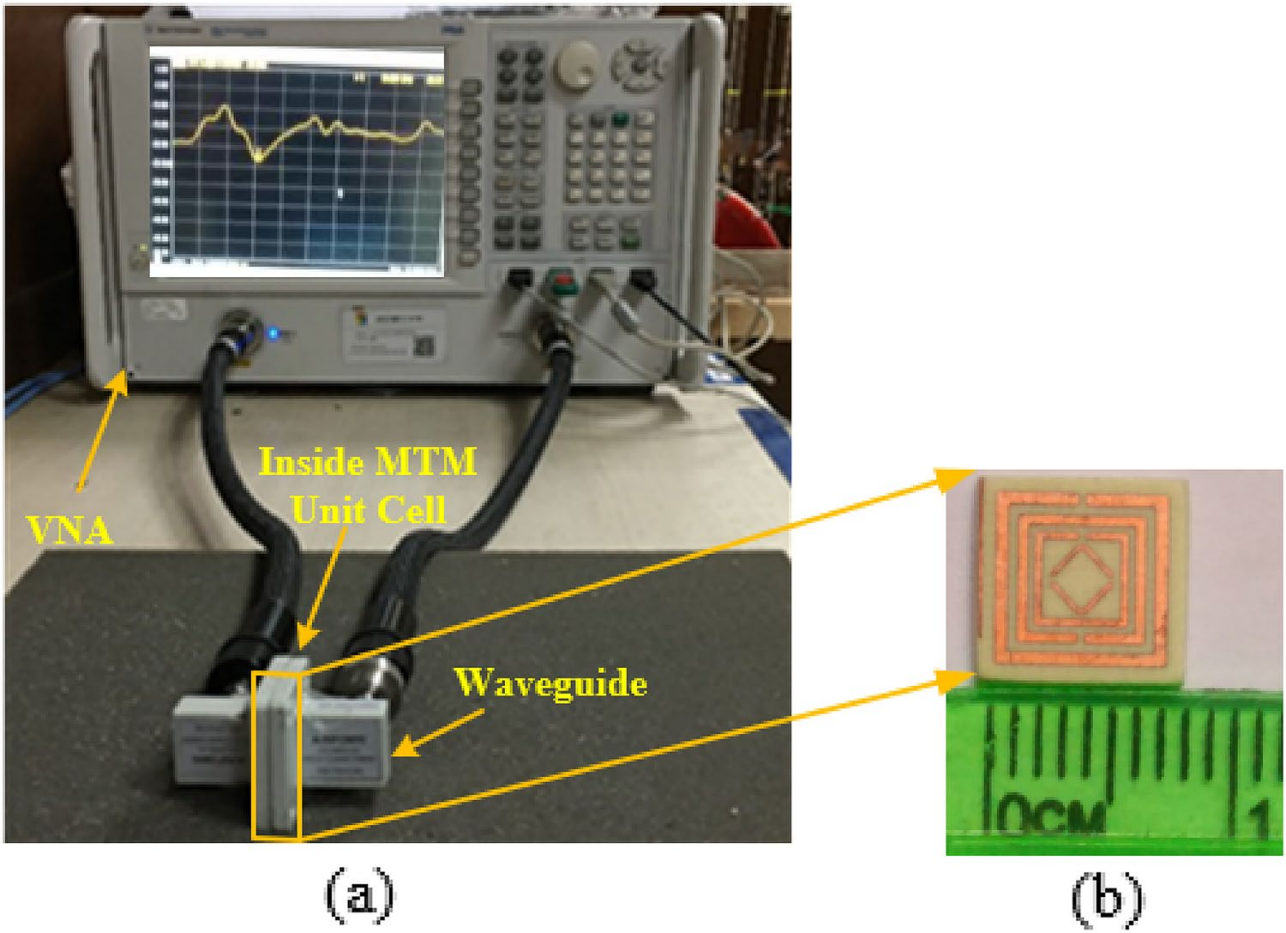
Unit cell (**a**) measurement setup (**b**) fabricated prototype size of 8 $\times$ 8 mm^2^.

**Figure 20:**
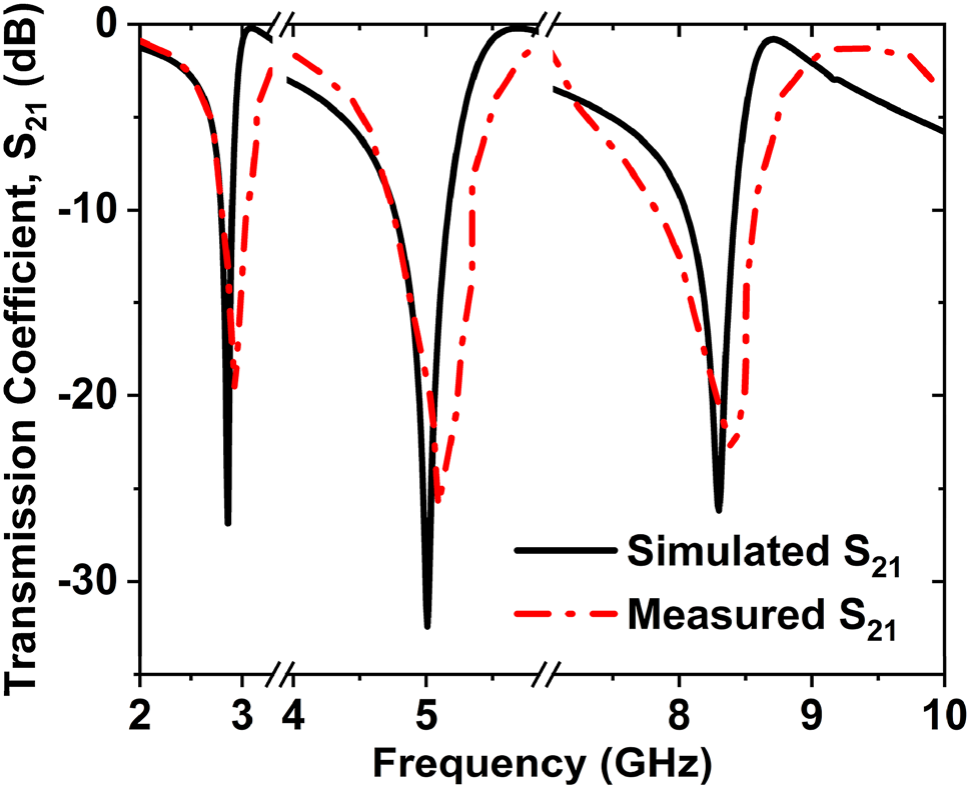
**S**imulated and measured transmission coefficient (S_21_) for the double inverse V-shaped unit cell.

Figure 19a,b depicts the MTM unit cell measurement setup and the unit cell fabricated prototype. At first, the vector network analyzer (VNA model N5227A) of the PNA series was calibrated by the Agilent N4694-60001 calibration kit. The VNA is connected to the two waveguides by the coaxial cables. One waveguide acts as a transmitter, and another one acts as a receiver. To measure, the fabricated prototype is placed in between two waveguides using the close boundary condition. The waveguide models 340WCAS, 187WCAS, and 112WCAS were utilized for the frequency ranges of 2.20–3.30 GHz, 3.95–5.85 GHz, and 7.05–10 GHz, respectively and a total frequency range of 2–10 GHz. The values of S_21_ were taken from the VNA as a prn form, then we have processed the prn data and made the S_21_ graph. Figure 20 shows the measured and simulated S_21_ graph which has a close similarity to each other. The measured resonance frequencies of S_21_ are 2.87, 5.01, and 8.32 GHz with magnitudes of − 26.86, − 32.42, and − 26.16 dB, respectively. The measured − 10 dB bandwidth of S_21_ are 2.80 to 2.91, 4.76 to 5.17, and 8.05 to 8.42 GHz. The simulated and measured S_21_ results were slightly different for the following factors. The Agilent N5227A VNA's calibration inaccuracy is one of the causes for the differences between the two procedures. A little difference occurred during the production of the Rogers RO4350B substrate layer. Furthermore, the substrate material is significant in the production of S-parameters. Waveguides coupling effect, the permittivity of the dielectric substrate also influences the resonance frequency. Capacitance values alter as a result of the dielectric constant, which is also responsible for adjusting the resonance frequency.

**Figure 21:**
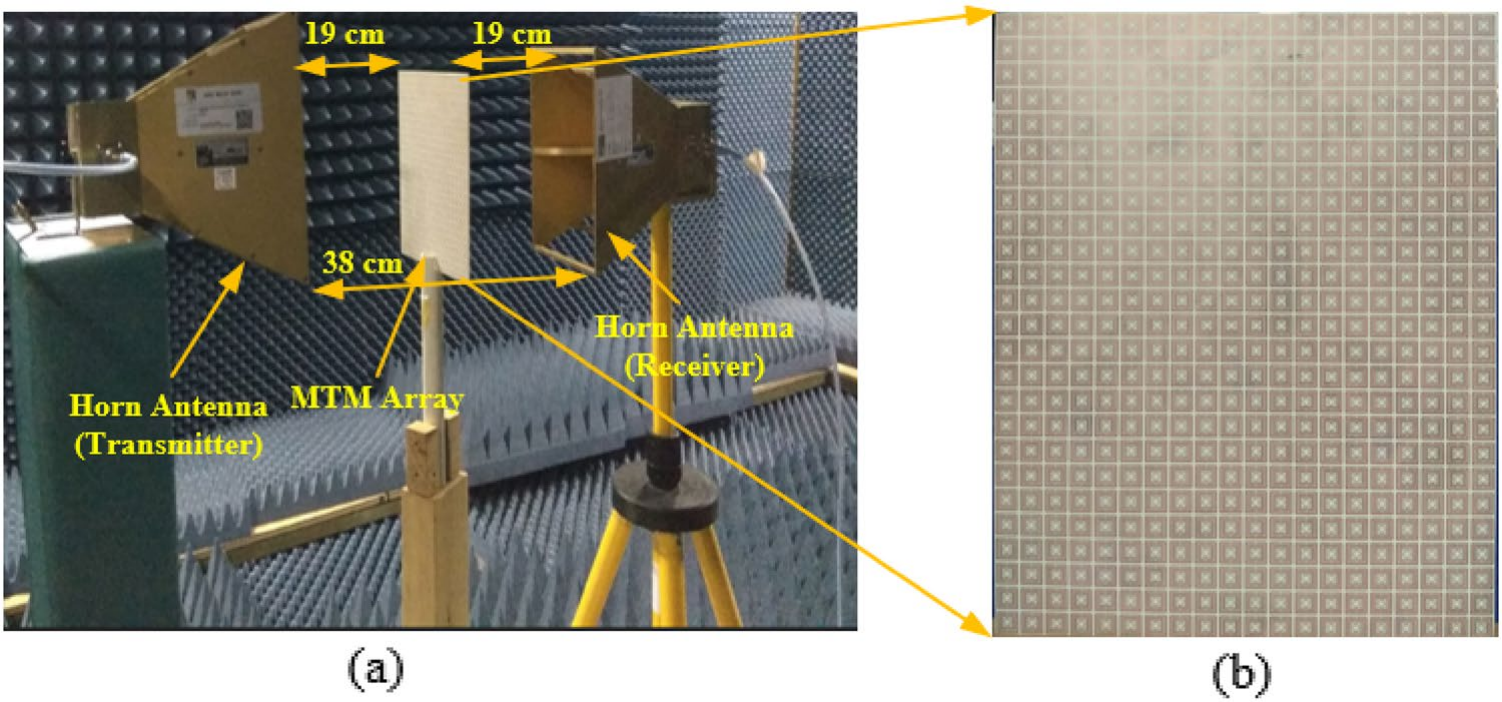
MTM array (**a**) measurement setup (**b**) fabricated prototype size of 160 $\times$ 200 mm^2^.

**Figure 22:**
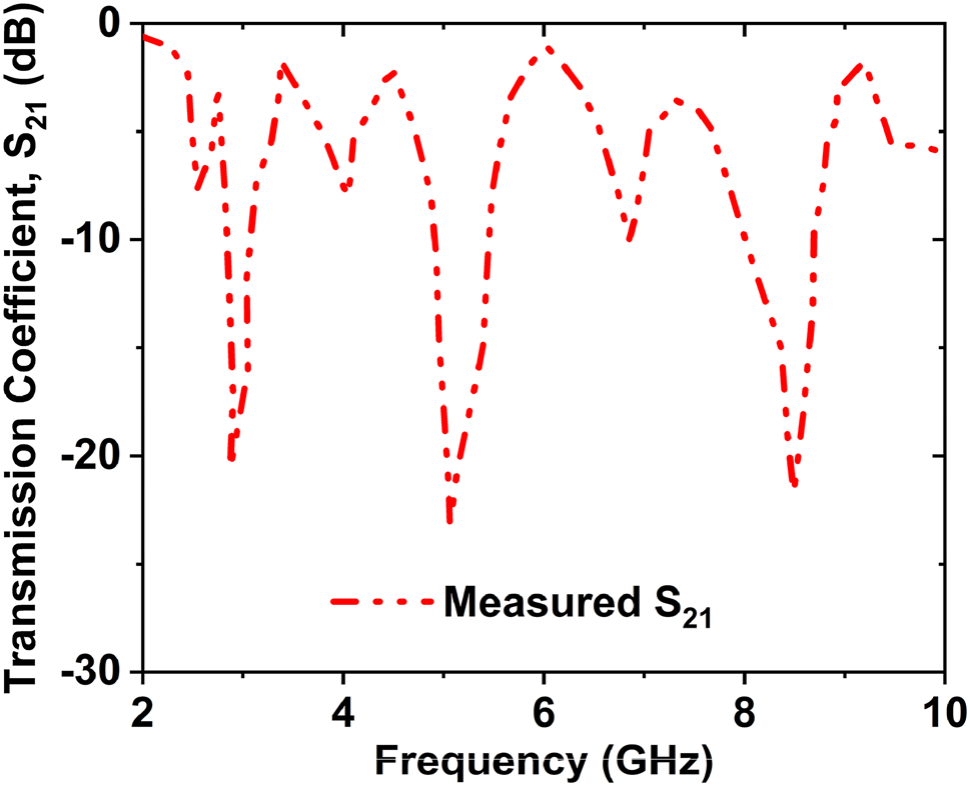
Transmission response (S_21_) for measured results of the 20 $\times$ 25 array structure.

Figure 21a,b reveals the array of the MTM unit cell measurement setup and fabricated MTM 20 $\times$ 25 array prototype. For the MTM array measurement, the developed prototype was placed in between two horn antennas, and these antennas were connected with VNA by the lossy coaxial cable. One horn antenna acts as a transmitter, and another one acts as a receiver. 2–10 GHz frequency range is used to take the S_21_ results of the array. When measured the array structure, we took 201 data points, i.e., in VNA, we set the number of data points to 201. The distance between the two horn antennas is 38 cm. For normal incidences, the wave propagates in the z-direction. Horn antennas were placed in an anechoic chamber to avoid the outer noise. The values of S_21_ were taken from the VNA as a prn form, then we have processed the prn data and made the S_21_ graph. Figure 22 shows the measured frequency versus S_21_ curve for the 20 $\times$ 25 array of the structure. There are some additional resonance frequencies in the measurement data due to the mutual coupling effects of the unit cell architectures, the coupling capacitor effect of the prototype. Due to the long-extended wire from the horn antenna to the VNA, the prototype's restricted dimension relative to the horn antenna causes some measurement errors, and the measured data contain some noise and harmonics.

The designed metamaterial structure provides three resonance frequencies are 2.86, 5, and 8.30 GHz, it covers S-, C-, and X-bands. Wi-Fi (wireless fidelity) is a widely used wireless networking protocol. Wi-Fi works in the same way as other wireless devices in that it sends signals between devices using radio frequencies. It works in 2.4 and 5 GHz frequency bands^50^. Wi-Fi network speeds are generally faster when frequencies in the 5 GHz band are used. The 5 GHz band has a smaller coverage area, but it delivers data quicker and has difficulty penetrating solid things. Higher frequencies allow data to be delivered quicker than lower frequencies; hence the 5 GHz band allows for faster uploading and downloading of information. Since the proposed structure provides a 5 GHz frequency band, the suggested MTM can be used in Wi-Fi for high bandwidth and high-speed applications. The marine radar is possibly the most important piece of equipment on the ship's bridge for the officer on watch (OOW) to keep a safe navigating watch. The X-band is extensively utilized in marine radars because it allows smaller antennas that fit on most boats and provides improved target resolution. A weather radar is a device that sends pulses of electromagnetic electricity into the ecosystem to locate precipitation, decides its movement and intensity, and discover the precipitation kind which includes rain, snow, or hail. The S-Band radar provides the ultimate long-range perspective, allowing to plan, forecast, and defend before severe weather. Since the designed cell provides more two resonance frequencies i.e., 2.86 GHz (S-band) and 8.30 GHz (X-band), so the proposed metamaterial could be used in weather radar and marine radar^51^.

The following steps can solve the loss problem of the radar and Wi-Fi. The power of the transmitter and the length of the MTM have an impact on the widest range of radar systems. The radar system's performance improves as transmission power and MTM size increase. The unavoidable noise appears as its input influences the receiver's ability to perform according to its specifications. Because of the noise generated by the gadget, the frequencies attributed to the microwave radar, for example, may go undetected. Frequency interference can potentially be resolved by altering the Wi-Fi router's channel. The broadcast frequency can usually be set for the channel. The more expensive and powerful routers can broadcast at a frequency of 5 GHz, which is fantastic.

## EMR analysis

The effective medium ratio (EMR) is an important consideration in the MTM study. The EMR represents the metamaterial's smallness and effectiveness^37^. Many devices work in the low resonance frequency, but the low resonance frequency is challenged to achieve for the small size of metamaterial; the high EMR represents the perfectness and criteria fulfil of the MTM design. If the value of EMR is less than 4, then the criterion of subwavelength of metamaterial is not fulfilled. The frequency and size of the metamaterial are inversely proportional to each other, the small size of MTM provides a high resonance frequency which is not applicable for low-frequency devices. So, it is necessary to consider EMR when designing metamaterial. The EMR is the ratio between the wavelength and unit cell size, presented by the Eq. (14).

14 $$EMR= \lambda /L$$

$\lambda$ = wavelength at the lower resonance frequency and $L$= length of the MTM unit cell.

The advantages of high EMR are small metamaterial size with low frequency, which is applicable for low-frequency devices. Furthermore, the high EMR demonstrates that the proposed design fulfils the MTM criteria. The proposed MTM unit cell has a high EMR of 13.1 for a size of 8 $\times$ 8 $\times$ 1.524 mm^3^ and a lower resonance frequency of 2.86 GHz. The suggested unit cell's EMR value of 13.11 increases its consistency while reducing its electrical dimensions without imposing fabrication constraints. The important advantages of the high EMR are: (i) enhanced uniformity of the properties, (ii) decreased electrical length without fabrication limit, and (iii) diminished effects of coupling with low transmission^22^.

Table 3 compares the proposed MTM unit cell structure with the existing unit cell structures. According to the research, the proposed unit cell structure covers the tri-band frequencies with double negative MTM properties. From the references^19,23,24,28,29,31–33,38,39,41^, it is seen that the unit cell size is large, covering frequency band, and EMR is lower than the proposed inverse double V-shaped unit cell. In addition, from the references^17,22,42^, we can see that the unit cell size is large, EMR is lower than the recommended unit cell, though the covering band is equal. Thus, the overall performance of the suggested unit cell is superior to that of the structure mentioned in Table 3.

**Table 3:** Comparison between suggested and existing work based on some aspects.

References	Published Year	Shape of the MTM Unit Cell	Unit Cell Size (mm^2^)	Frequency Bands	MTM types	EMR
^17^	2020	Concentric Crossed Line	10 $\times$ 10	C-, X-, Ku-	SNG	4.5
^19^	2016	Modified Z	10 $\times$ 10	X-	DNG	4
^22^	2017	Double C	12 $\times$ 12	S-, C-, X-	DNG	7.44
^23^	2016	Double Z-Shaped	8.5 $\times$ 8.5	C-, X-	DNG	4.8
^24^	2019	U-joint double split O	15 $\times$ 12	X-, Ku-	DNG	4.5
^28^	2019	Resistor Loaded Sector	12.5 $\times$ 12.5	C-, X-	SNG	NR
^29^	2015	S-Shaped	12 $\times$ 12	X-, Ku-	SNG	2.09
^31^	2017	Four-Fold Symmetric	28.2 $\times$ 28.2	C-	SNG	NR
^32^	2019	Nickel Concentrated	25 $\times$ 20	X-, Ku-	DNG	NR
^33^	2020	Hexagonal	10 $\times$ 10	S-, X-	DNG	8.4
^38^	2018	Modified H	9 $\times$ 9	X-, Ku-	DNG	3.0
^39^	2017	S-Shaped	10 $\times$ 10	X-	SNG	2.4
^41^	2018	Circular Sector	9 $\times$ 9	C-	SNG	NR
^42^	2020	Circular CSRR Shaped	9 $\times$ 9	S-, C-, X-	SNG	9.52
This work	2021	Inverse double V-Shaped	8 $\times$ 8	S-, C-, X-	DNG	13.11

*NR not reported.

## Conclusion

In this article, a DNG metamaterial has been established and investigated numerically and experimentally for radar and Wi-Fi applications. The designed metamaterial delivers three transmission resonance frequencies such as 2.86, 5, and 8.30 GHz, covering S-, C-, and X-bands. The entire dimension of the suggested unit cell is 8 $\times$ 8 $\times$ 1.524 mm^3^, and a high EMR value of 13.11 is found from this structure. Since the proposed MTM is DNG, so the DNG region has been found from 2.82 to 2.89, 5.04 to 5.23, and 8.36 to 8.46 GHz. The S-band (2.86 GHz) is applied in, weather radar. The marine radar works in X- band (8.30 GHz). The C-band (5 GHz) is widely used in Wi-Fi for high bandwidth and high-speed applications. Wi-Fi was originally designed for mobile computing devices such as laptops, but it is now widely used in consumer goods such as televisions, DVD players, and digital cameras. Parametric tests on various lengths and widths with various structures have been used to determine the effective medium parameters. Different types of array structure 1 $\times$ 2, 2 $\times$ 2, 3 $\times$ 3, 4 $\times$ 4, and 20 $\times$ 25 is analyzed and validated by the measured result. The simulated and measured result is a good deal for the inverse double V-shaped unit cell and an array of the cell. We got the indicated formation's whole performance better than the other structures mentioned in this paper. Since the proposed metamaterial unit cell size is small, provides desired resonance frequency, and gives a large frequency band and high EMR value; hence the suggested metamaterial can be effectively used in radar and Wi-Fi applications.

## References

[1] Caloz C, Itoh T (2005). Electromagnetic Metamaterials: Transmission Line Theory and Microwave Applications.

[2] Liu R, Ji C, Zhao Z, Zhou T (2015). Metamaterials: Reshape and rethink. Engineering.

[3] Viktor G (1968). The electrodynamics of substances with simultaneously negative values of ε and μ. Soviet Phys. Uspekhi.

[4] Islam M, Islam MT, Samsuzzaman M, Faruque MRI (2015). Compact metamaterial antenna for UWB applications. Electron. Lett..

[5] Misran N, Yusop SH, Islam MT, Ismail MY (2012). Analysis of parameterization substrate thickness and permittivity for concentric split ring square reflectarray element. J. Kejuruter. (J. Eng.).

[6] Zheng, Z., Wang, W., Zhang, H.-T., Zheng, Y.-Y. & Liang, X.-L. Dual-band anti-interference slot antenna using metamaterial structure. In *2019 IEEE International Symposium on Antennas and Propagation and USNC-URSI Radio Science Meeting* 327–328 (2019).

[7] Il Kwak S, Sim D-U, Kwon JH, Yoon YJ (2016). Design of PIFA with metamaterials for body-SAR reduction in wearable applications. IEEE Trans. Electromagn. Compat..

[8] Alitalo P, Tretyakov S (2009). Electromagnetic cloaking with metamaterials. Mater. Today.

[9] Chen M, Jiang H, Zhang H, Li D, Wang Y (2018). Design of an acoustic superlens using single-phase metamaterials with a star-shaped lattice structure. Sci. Rep..

[10] Islam MR, Islam MT, Moniruzzaman M, Samsuzzaman M, Arshad H (2021). Penta band single negative meta-atom absorber designed on square enclosed star-shaped modified split ring resonator for S-, C-, X-and Ku-bands microwave applications. Sci. Rep..

[11] Deng G, Lv K, Sun H, Yang J, Yin Z, Li Y (2020). An ultrathin, triple-band metamaterial absorber with wide-incident-angle stability for conformal applications at x and ku frequency band. Nanoscale Res. Lett..

[12] Liu W, Sun H, Xu L (2018). A microwave method for dielectric characterization measurement of small liquids using a metamaterial-based sensor. Sensors.

[13] Abdulkarim YI, Deng L, Luo H, Huang S, Karaaslan M, Altıntaş O (2020). Design and study of a metamaterial based sensor for the application of liquid chemicals detection. J. Market. Res..

[14] Islam MR, Samsuzzaman M, Misran N, Beng GK, Islam MT (2020). A tri-band left-handed meta-atom enabled designed with high effective medium ratio for microwave based applications. Results Phys..

[15] Islam MR, Islam MT, Moniruzzaman M, Samsuzzaman M, Bais B, Arshad H (2020). Square enclosed circle split ring resonator enabled epsilon negative (ENG) near zero index (NZI) metamaterial for gain enhancement of multiband satellite and radar antenna applications. Results Phys..

[16] Islam SS, Rahman MA, Faruque MRI, Islam MT (2018). Design and analysis with different substrate materials of a new metamaterial for satellite applications. Sci. Eng. Compos. Mater..

[17] Islam MS, Islam MT, Sahar NM, Rmili H, Amin N, Chowdhury ME (2020). A mutual coupled concentric crossed-Line split ring resonator (CCSRR) based epsilon negative (ENG) metamaterial for Tri-band microwave applications. Results Phys..

[18] Hossain M, Faruque MRI, Islam MT, Islam S (2017). "An effective medium ratio obeying meta-atom for multiband applications. Bull. Tech. Pol. Acad. Sci..

[19] Hasan M, Faruque MRI, Islam SS, Islam MT (2016). A new compact double-negative miniaturized metamaterial for wideband operation. Materials.

[20] Cheng Y, Zou Y, Luo H, Chen F, Mao X (2019). Compact ultra-thin seven-band microwave metamaterial absorber based on a single resonator structure. J. Electron. Mater..

[21] Chen F, Cheng Y, Luo H (2020). A broadband tunable terahertz metamaterial absorber based on single-layer complementary gammadion-shaped graphene. Materials.

[22] Hossain MJ, Faruque MRI, Islam MT (2017). Design and analysis of a new composite double negative metamaterial for multi-band communication. Curr. Appl. Phys..

[23] Zhou H, Wang C, Peng H (2016). A novel double-incidence and multi-band left-handed metamaterials composed of double Z-shaped structure. J. Mater. Sci.: Mater. Electron..

[24] Hoque A, Islam MT, Almutairi AF, Faruque MRI, Singh MJ, Islam MS (2019). U-joint double split O (UDO) shaped with split square metasurface absorber for X and ku band application. Results Phys..

[25] Hossain A, Islam MT, Misran N, Islam MS, Samsuzzaman M (2021). A mutual coupled spider net-shaped triple split ring resonator based epsilon-negative metamaterials with high effective medium ratio for quad-band microwave applications. Results Phys..

[26] Islam SS, Faruque MRI, Islam MT (2014). The design and analysis of a novel split-H-shaped metamaterial for multi-band microwave applications. Materials.

[27] Cheng Y, Luo H, Chen F (2020). Broadband metamaterial microwave absorber based on asymmetric sectional resonator structures. J. Appl. Phys..

[28] Kalraiya S, Chaudhary RK, Abdalla MA (2019). Design and analysis of polarization independent conformal wideband metamaterial absorber using resistor loaded sector shaped resonators. J. Appl. Phys..

[29] Zhou Z, Yang H (2015). Triple-band asymmetric transmission of linear polarization with deformed S-shape bilayer chiral metamaterial. Appl. Phys. A.

[30] Islam MT, Islam MR, Islam MT, Hoque A, Samsuzzaman M (2021). Linear regression of sensitivity for meander line parasitic resonator based on ENG metamaterial in the application of sensing. J. Market. Res..

[31] Thummaluru SR, Mishra N, Chaudhary RK (2017). Design and analysis of an ultrathin triple-band polarization independent metamaterial absorber. AEU-Int. J. Electron. Commun..

[32] Faruque MRI, Ahamed E, Rahman MA, Islam MT (2019). Flexible nickel aluminate (NiAl2O4) based dual-band double negative metamaterial for microwave applications. Results Phys..

[33] Islam MS, Samsuzzaman M, Beng GK, Misran N, Amin N, Islam MT (2020). A gap coupled hexagonal split ring resonator based metamaterial for S-band and X-band microwave applications. IEEE Access.

[34] Rao MV, Madhav B, Anilkumar T, Nadh BP (2018). Metamaterial inspired quad band circularly polarized antenna for WLAN/ISM/Bluetooth/WiMAX and satellite communication applications. AEU-Int. J. Electron. Commun..

[35] Cheng Y, Fan J, Luo H, Chen F (2019). Dual-band and high-efficiency circular polarization convertor based on anisotropic metamaterial. IEEE Access.

[36] Zhou E, Cheng Y, Chen F, Luo H (2021). Wideband and high-gain patch antenna with reflective focusing metasurface. AEU-Int. J. Electron. Commun..

[37] Hossain MJ, Faruque MRI, Islam MT (2018). Effective medium ratio obeying wideband left-handed miniaturized meta-atoms for multi-band applications. J. Electron. Mater..

[38] Hossain TM, Jamlos MF, Jamlos MA, Soh PJ, Islam MI, Khan R (2018). Modified H-shaped DNG metamaterial for multiband microwave application. Appl. Phys. A.

[39] Kumari K, Mishra N, Chaudhary RK (2017). A n ultra-thin compact polarization insensitive dual band absorber based on metamaterial for X-band applications. Microw. Opt. Technol. Lett..

[40] Almutairi AF, Islam MS, Samsuzzaman M, Islam MT, Misran N, Islam MT (2019). A complementary split ring resonator based metamaterial with effective medium ratio for C-band microwave applications. Results Phys..

[41] Thummaluru SR, Chaudhary RK (2018). Polarization controllable and wide-angle frequency tunable metamaterial absorber. J. Appl. Phys..

[42] Venkateswara Rao M, Madhav BTP, Anilkumar T, Prudhvinadh B (2020). Circularly polarized flexible antenna on liquid crystal polymer substrate material with metamaterial loading. Microwave Opt. Technol. Lett..

[43] CST AG, D. *CST Studio Suite*. https://www.3ds.com/products-services/simulia/products/cst-studio-suite/ (2019).

[44] Luukkonen O, Maslovski SI, Tretyakov SA (2011). A stepwise Nicolson–Ross–Weir-based material parameter extraction method. IEEE Antennas Wirel. Propag. Lett..

[45] PathWave Advanced Design System (ADS). https://www.keysight.com/sg/en/lib/resources/software-releases/pathwave-ads-2019.html (2019).

[46] Paul CR (2011). Inductance: Loop and Partial.

[47] Wartak MS, Tsakmakidis KL, Hess O (2011). Introduction to metamaterials. Phys. Can..

[48] Hasan MM, Faruque MRI, Islam MT (2018). Beam steering of eye shape metamaterial design on dispersive media by FDTD method. Int. J. Numer. Model..

[49] Ziolkowski RW (2003). Design, fabrication, and testing of double negative metamaterials. IEEE Trans. Antennas Propag..

[50] Hannan S, Islam MT, Faruque MRI, Chowdhury ME, Musharavati F (2021). Angle-insensitive co-polarized metamaterial absorber based on equivalent circuit analysis for dual band WiFi applications. Sci. Rep..

[51] Brookner, E. Metamaterial advances for radar and communications. In *2016 CIE International Conference on Radar (RADAR)* 1–8 (2016).

